# Inter-individual heterogeneity of functional brain networks in children with autism spectrum disorder

**DOI:** 10.1186/s13229-022-00535-0

**Published:** 2022-12-26

**Authors:** Xiaonan Guo, Guangjin Zhai, Junfeng Liu, Yabo Cao, Xia Zhang, Dong Cui, Le Gao

**Affiliations:** 1 School of Information Science and Engineering, Yanshan University, Qinhuangdao, 066004 China; 2 Hebei Key Laboratory of Information Transmission and Signal Processing, Yanshan University, Qinhuangdao, 066004 China; 3 Department of Neurology, West China Hospital, Sichuan University, Chengdu, 610041 China

**Keywords:** Autism spectrum disorder, Functional magnetic resonance imaging, Functional connectivity, k-means clustering, Subtype

## Abstract

**Background:**

Autism spectrum disorder (ASD) is a neurodevelopmental disorder with considerable clinical heterogeneity. This study aimed to explore the heterogeneity of ASD based on inter-individual heterogeneity of functional brain networks.

**Methods:**

Resting-state functional magnetic resonance imaging data from the Autism Brain Imaging Data Exchange database were used in this study for 105 children with ASD and 102 demographically matched typical controls (TC) children. Functional connectivity (FC) networks were first obtained for ASD and TC groups, and inter-individual deviation of functional connectivity (IDFC) from the TC group was then calculated for each individual with ASD. A k-means clustering algorithm was used to obtain ASD subtypes based on IDFC patterns. The FC patterns were further compared between ASD subtypes and the TC group from the brain region, network, and whole-brain levels. The relationship between IDFC and the severity of clinical symptoms of ASD for ASD subtypes was also analyzed using a support vector regression model.

**Results:**

Two ASD subtypes were identified based on the IDFC patterns. Compared with the TC group, the ASD subtype 1 group exhibited a hypoconnectivity pattern and the ASD subtype 2 group exhibited a hyperconnectivity pattern. IDFC for ASD subtype 1 and subtype 2 was found to predict the severity of social communication impairments and the severity of restricted and repetitive behaviors in ASD, respectively.

**Limitations:**

Only male children were selected for this study, which limits the ability to study the effects of gender and development on ASD heterogeneity.

**Conclusions:**

These results suggest the existence of subtypes with different FC patterns in ASD and provide insight into the complex pathophysiological mechanism of clinical manifestations of ASD.

**Supplementary Information:**

The online version contains supplementary material available at 10.1186/s13229-022-00535-0.

## Introduction

Autism spectrum disorder (ASD) is an early-onset neurodevelopmental disorder. Its core symptoms are social interaction and communication impairments, repetitive, stereotyped behaviors, and narrow interests [1]. The etiology of ASD is unclear, and current research generally indicates that the clinical presentation of individuals with ASD is highly heterogeneous [2–4]. Due to the high clinical heterogeneity of ASD, the diagnostic criteria have evolved over the years and now include a significantly broader spectrum [5]. Therefore, the interpretation of the heterogeneity pattern of ASD is a pressing issue.

There is a general consensus that ASD is related to an atypical pattern of brain functional networks [6, 7]. Abnormal resting-state brain functional connectivity (FC) patterns in ASD have been successively identified in various brain regions and networks, such as the amygdala, visual network, and default-mode network [8–10]. Both reduced and increased patterns of FC in the brain of individuals with ASD have been reported, which are associated with developmental stage [7, 11–14]. Although studies continue to report atypical FC in the brain of individuals with ASD, the results are often inconsistent [15], which hinders the study of neurobiological mechanisms of ASD and the identification of effective biomarkers to guide clinical diagnosis and treatment of ASD.

Studying inter-individual differences in brain networks is essential for understanding complex psychiatric disorders [16]. For example, studies of inter-individual differences in functional brain networks have found that it can be used to predict schizophrenia positive symptoms [17]. Highly heterogeneous brain structures are reported in ASD, and there is a link between heterogeneity of brain structures and clinical symptoms, which together influence the ASD classification [18]. In addition, subtyping of ASD is a direction to study the high heterogeneity of ASD. Performance of supervised learning for prediction of Autism Diagnostic Observation Schedule (ADOS) scores can be improved by differentiating multidimensional neuroanatomical subtypes [19]. ASD subtype’s unique brain–behavior relationships were reported in the FC-based ASD subtype study [20]. ASD subtyping studies have been widely applied to analyze the functional and structural networks of the ASD brain and have explained to some extent the heterogeneity of ASD [16]. However, to the best of our knowledge, existing studies on ASD subtypes in the field of ASD functional brain networks still distinguish subtypes solely rely on ASD brain network characteristics, ignoring the brain network information provided by the typical controls (TC) group. Previous studies have suggested that the heterogeneity of atypical FC patterns in ASD stems from idiosyncratic distortions of the FC pattern [21]. Therefore, subtyping studies based on inter-individual deviation could better reveal the heterogeneous pattern of individuals with ASD.

This study aimed to explore the heterogeneity pattern of brain functional network among individuals with ASD based on the inter-individual deviation between ASD and TC. Based on the FC network for each individual, the inter-individual deviation of functional connectivity (IDFC) of each individual with ASD from the TC group is calculated. Individuals with ASD were then clustered based on IDFC to obtain ASD subtypes. The relationship between IDFC and clinical symptoms of ASD was also assessed. We predict that individuals with ASD have different functional network patterns of IDFC and can be subdivided into subtypes based on the IDFC.

## Materials and methods

### Participants

Resting-state functional magnetic resonance imaging (fMRI) and phenotype data from the open-access Autism Brain Imaging Data Exchange database (ABIDE, fcon_1000.projects.nitrc.org/indi/abide/) were used [22]. The participant selection principles were the same as in our previous study [23]. Exclusion criteria include: (a) subjects younger than 7 years of age or older than 12 years of age; (b) female subjects (female subjects were less than 10% of the total data set); (c) subjects with excessive head motion during resting-state scanning (i.e., motion greater than 2 mm translation and 2 degrees of rotation, and greater than 50% of frames with large frame-wise displacement [FD]); (d) subjects with missing information on full intelligence quotient (FIQ), handedness or eye status values; and (e) subjects with low-quality scanned structural images. The remaining subjects maximized *p* values for group differences in age, handedness, FIQ, eye status, and mean FD by using a data-driven algorithm to create well-matched datasets of ASD and TC groups within each site. Data from centers with fewer than 10 subjects per group were finally removed. The final remaining 105 children with ASD and 102 TC from 6 centers were applied to the study. Participant demographic details are summarized in Table 1.

**Table 1:** Demographics of the participants

	ASD (*n* = 105)	TC (*n* = 102)	*p* value
Age (years)	10.15 ± 1.26	10.02 ± 1.38	0.48^a^
FIQ	110.53 ± 17.42	113.78 ± 11.92	0.12^a^
Mean FD (mm)	0.17 ± 0.08	0.16 ± 0.08	0.16^a^
Eye status (open/closed)	91/14	88/14	0.93^b^
Handedness (right/left/mixed)	83/9/13	82/5/15	0.54^b^
ADOS_COMM	3.13 ± 1.59	–	–
ADOS_SOCIAL	8.01 ± 2.51	–	–
ADOS_RRB	2.23 ± 1.63	–	–

*ASD* Autism spectrum disorder, *TC* Typical controls, *FIQ* Full-scale intelligence quotient, *FD* Frame-wise displacement, *ADOS* Autism Diagnostic Observation Schedule (available for 82 ASD subjects), *ADOS_COMM* Communication subscore of the ADOS, *ADOS_SOCIAL* Social subscore of the ADOS, *ADOS_RRB* Stereotyped behaviors and restricted interests subscore of the ADOS

^a^Two-sample t tests were used to compare age, FIQ, and mean FD parameters between the two groups

^b^*χ*2 tests were used to compare eye status and handedness

### Data preprocessing

The advanced edition of Data Processing Assistant for Resting-State fMRI (DPARSF A, http://rfmri.org/DPARSF) toolbox [24] was used to preprocess the resting-state fMRI data of the subjects. The same preprocessing steps as in our previous study were used [23]. Main steps include: the first 10 volumes of each subject being removed, slicing time corrected, spatial realigned (participants with translational or rotational motion higher than 2 mm or 2° were excluded), normalized to standard Montreal Neurological Institute (MNI) stereotaxic space, and resampling to 3 × 3 × 3 mm^3^, spatial smoothing with an isotropic Gaussian kernel (full width at half maximum = 6 mm), linear trends were removed, potential motion artifacts were resolved by using the 3dDespike algorithm in Analysis of Functional NeuroImaging (https://afni.nimh.nih.gov/afni/), potential nuisance signals were regressed (Friston-24 motion parameters, white matter, and cerebrospinal fluid signals) [25–27], and bandpass filtering was carried out at 0.01–0.1 Hz. In addition, to reduce the effect of head movement on the FC analysis [28], we examined the percentage of high head movement time points for each subject. Time points with a mean head movement displacement greater than 0.5 mm and their preceding and following two time points were marked as high head movement time points [29]. Subjects with more than 50% high head movement time points were not included in the following analysis [30, 31].

### Inter-individual deviation of functional connectivity

The time series data were extracted according to a 264-region of interest (ROI) partitioning scheme, and 10 functional networks were delineated [32, 33]. The functional networks include the motor and somatosensory network (SMN), cingulo-opercular network (CO), auditory network (AN), default-mode network (DMN), visual network (VN), fronto-parietal network (FPN), salience network (SAN), subcortical network (SUB), ventral attention network (VAN), and dorsal attention network (DAN). ROIs that are not part of the 10 functional networks are included in the uncertain network (UND). A 264 × 264 Pearson correlation matrix was constructed for each subject as a measure of inter-regional FC, and we set the negative correlation to zero due to the current ambiguity about the meaning of negative correlation [34, 35]. To prevent skewing of the data distribution due to zeroing of negative correlation, we tested the reliability of the results by retesting the results using the correlation matrix without removing negative correlation (see Additional file 1). The IDFC of subject *i* from the ASD group at ROI *k* is defined as:

$$V_{ik} = E\left\{ {1 - \frac{{F_{ik} F_{jk}^{\prime } }}{{\sqrt {(F_{ik} F_{ik}^{\prime } )(F_{jk} F_{jk}^{\prime } )} }}} \right\},j = 1,2, \ldots n$$

**Fig. 1:**
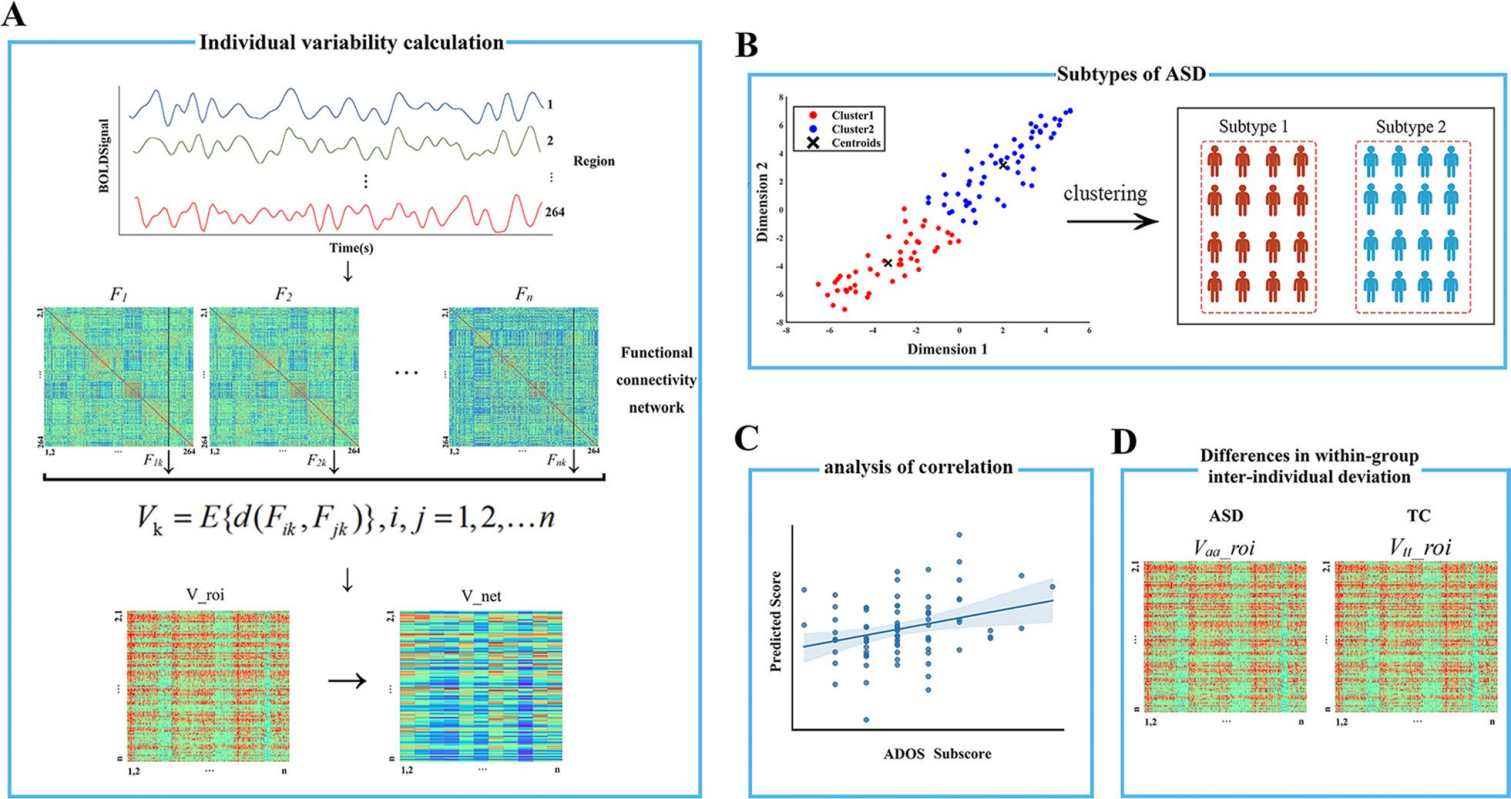
Flowchart of the inter-individual deviation of functional connectivity (IDFC) analysis. **A** Flowchart of IDFC matrix calculation. The IDFC matrix between the autism spectrum disorder (ASD) group and the typical controls (TC) group can be calculated from the functional connectivity (FC) network. *d*: the cosine distance between *F*_*ik*_ and *F*_*jk*_; V_roi: IDFC matrix at the region level; and V_net: IDFC matrix at the network level. **B** ASD subtypes were obtained by a k-means clustering analysis at the network level by using the IDFC at the network level as features. **C** A multivariate support vector regression model was used to analyze the relationship between network-level IDFC and severity of clinical symptoms of ASD. **D** The within-group inter-individual FC deviation of the ASD and TC groups was calculated and compared. *V*_*aa*_*_*roi: the matrix of inter-individual FC deviation within the ASD group at the region level; *V*_*tt*_*_*roi: the matrix of inter-individual FC deviation within the TC group at the region level

*F*_*ik*_ denotes the overall FC profile of brain region *k* of subject *i* with ASD defined by the functional architecture, i.e., *F*_*i*_(*k*,:). The vector *F*_*i*_(*k*,:) represents the *k*-th column of the correlation matrix of ASD subject *i*, which stores the correlation coefficients of brain region *k* with other brain regions. Similarly, *F*_*jk*_ denotes the overall FC profile of brain region *k* of subject *j* in the TC group. To characterize the functional deviations of ASD from the TC group, the cosine distances of the overall FC profile of region *k* between subject *i* from the ASD group and all subjects in the TC group were calculated and the mean value was used to describe the IDFC of ASD subject *i* at region *k*. At the region level, the IDFC matrix V_roi between the ASD and TC group was obtained by calculating the IDFC for all regions within the ASD group for each subject. The IDFC matrix V_net at the network level was obtained by averaging the IDFC of regions within the same network (Fig. 1A).

### Clustering analysis based on IDFC

We hypothesized the existence of subtypes of ASD with high heterogeneity of functional brain network characteristics and clustering analysis was performed on 105 individuals with ASD, as illustrated in Fig. 1B. The analysis was performed using the k-means clustering method, selecting network-level IDFC as the clustering feature, because analyzing the number of features from the region level greater than the number of subjects would lead to unreliable clustering results. The IDFC matrix V_net (a 105 × 11 matrix; 105 represents the number of ASD subjects, and 11 represents the number of networks) was calculated from the network level (including 10 functional networks and an uncertain network). A k-means clustering analysis was performed after regressing out the covariates (age, FIQ, mean FD, handedness, eye status, and sites) from the V_net matrix. For the optimal k value of clustering, it is determined by the mean silhouette value; that is, the silhouette of each point after clustering is calculated and the mean silhouette is calculated. The larger the mean silhouette is, the better the clustering effect is. The k-means clustering was repeated 19 times using k ranging from 2 to 20, and the mean silhouette value was calculated for each clustering analysis.

To determine whether clustering results were influenced by participant demographic characteristics, clinical symptom severity of ASD, and data sites, two-sample t tests were used to analyze whether there were significant differences in age, FIQ, mean FD, and ADOS scores between subtypes of ASD, χ2 tests were used to analyze whether there were significant differences in handedness and eye status between subtypes of ASD, and the distribution of sites was also examined for each subtype of ASD. To exclude the effect of potential heterogeneity among TC on the results, we randomly grouped individuals in the TC group to obtain two well-matched subgroups of TC (51 subjects in subgroup 1 and 51 subjects in subgroup 2). The cluster analysis was then replicated using the two TC subgroups to test for the reliability of this study (see Additional file 1).

### Atypical FC patterns of ASD subtypes

The obtained ASD subtypes and TC were compared for FC differences to determine whether there is an atypical FC pattern in the ASD subtypes. The FC differences between each ASD subtype group and TC group and between subtype groups were analyzed at three levels: region, network, and whole-brain. The FC matrix for each group was obtained by Fisher z-transformation of the Pearson’s correlation matrix for all subjects, and two-sample t tests were then performed to analyze the FC differences. Region and network-level results were false discovery rate (FDR) corrected, and statistical significance was set at *p* < 0.05. Results at the whole-brain level were corrected for Bonferroni (*q* < 0.05, *p* = 0.05/3 = 0.017). Age, FIQ, mean FD, handedness, eye status, and sites were used as covariates. At the network level, we examined the t-values of the two-sample t tests in the FC analysis of differences. And we calculated the percentage of connectivity edges with significant FC differences in ROI between networks to the total connectivity edges between networks based on the results of the FC difference analysis at the region level. These analyses were used to determine which networks had anomalies. The same methods were also used to analyze whether there were differences in FC between the whole ASD and TC groups at the region, network, and whole-brain levels.

### Relationship between IDFC of ASD subtypes and severity of clinical symptoms of ASD

For each ASD subtype, the relationship between the network-level IDFC (regressing out age, FIQ, mean FD, handedness, eye status, and sites as covariates), used to represent the degree of heterogeneity between individuals with ASD and TC, and the severity of ASD symptoms as assessed by ADOS subscores (i.e., communication, social, restricted and repetitive behaviors) was explored [36]. Only the 82 ASD subjects with complete ADOS subscale score information were used for the follow-up analysis. A multivariate support vector regression method was applied for the analysis, where the ADOS subscores were used as dependent variables and the IDFC of each network was used as the independent variable [37]. Library for Large Linear Classification (LIBLINEAR) (https://www.csie.ntu.edu.tw/~cjlin/liblinear/) toolbox was used in this analysis [38]. The regression analysis was performed using the L2-regularized support vector regression model. The performance of the regression algorithm was evaluated using leave-one-out cross-validation (LOOCV) [39]. Briefly, a model is constructed based on n-1 subjects and then a prediction is made for the remaining subject. After all subjects were predicted by the model, the correlation coefficient R between the predicted and observed values was calculated. Statistical significance was determined by a non-parametric permutation test [40], by disordering the labels to obtain a new correlation coefficient *R*_*p*_ based on the disordered dataset. The *p* value is determined by the ratio of the number of times the *R*_*p*_-value is greater than the *R*-value in 1000 permutations to the total number of permutations (i.e., 1000). To reflect the contribution of each network, we average the feature weights of each LOOCV as the feature weights of each network.

### Inter-individual FC deviation within the group

The FC heterogeneity within the group was also compared between ASD and TC groups. An algorithm similar to IDFC was used to represent the inter-individual FC deviation within the group. At the brain region level, the inter-individual FC deviation matrix *V*_*aa*_*_*roi for the ASD group can be obtained by using the FC of the ASD group as *F*_*ik*_ and *F*_*jk*_ in the IDFC calculation equation (*i* ≠ *j*). Similarly, the inter-individual FC deviation matrix *V*_*tt*_*_*roi for the TC group can be calculated. Two-sample t tests were performed on *V*_*aa*_*_*roi and *V*_*tt*_*_*roi to explore the patterns of differences. Age, FIQ, mean FD, handedness, eye status, and sites were used as covariates. The results were FDR corrected, and the statistical significance was set at *p* < 0.05.

## Results

### Subtyping ASD based on IDFC

**Fig. 2:**
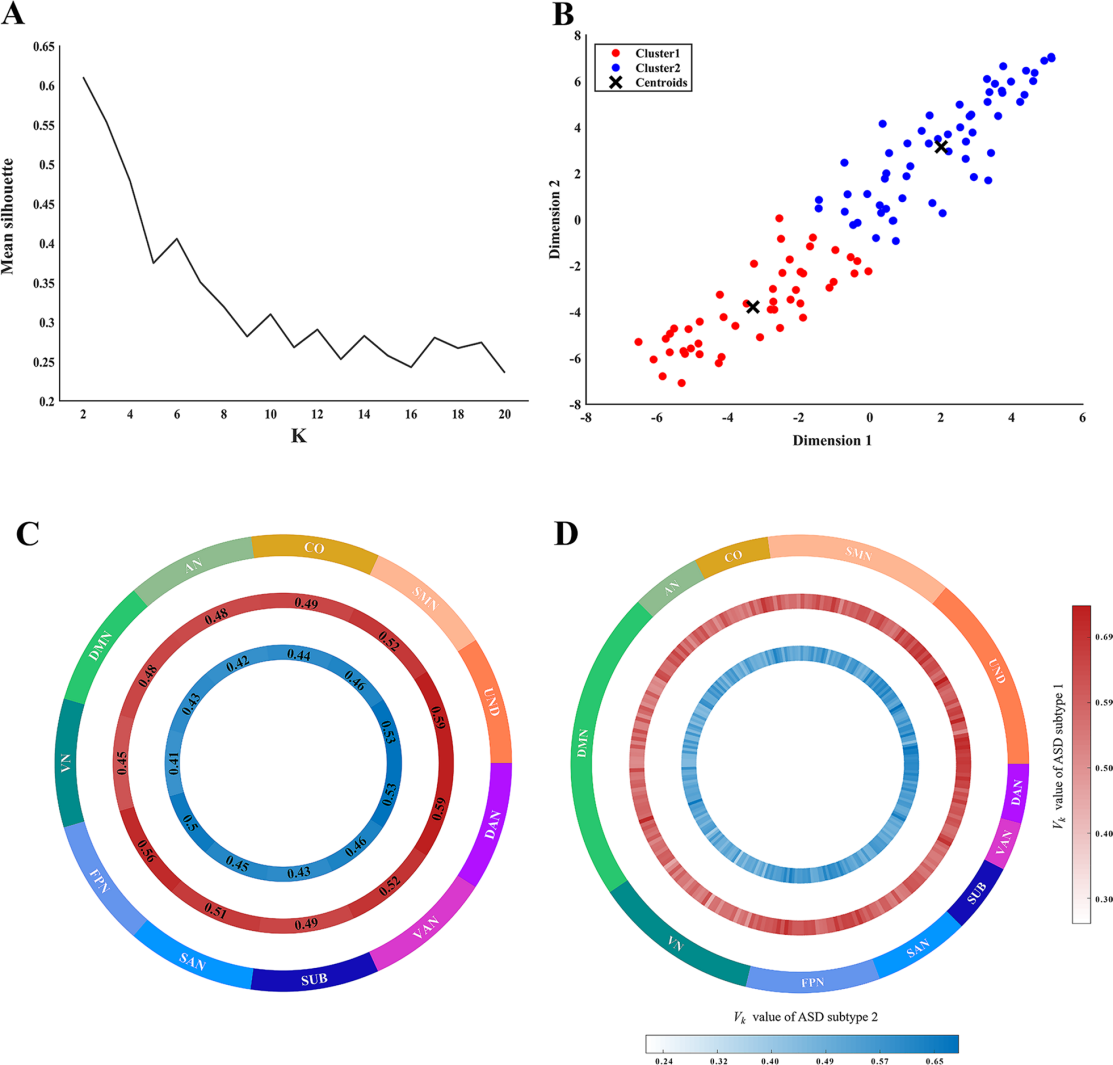
ASD subtype analysis based on the network-level IDFC. **A** Mean silhouette plots, indicating that the optimal number of clusters is 2. **B** The results of k-means clustering shown on the 2-dimensional plane. **C** Mean IDFC patterns at the network level of two ASD subtypes. Red inner ring represents ASD subjects belonging to subtype 1, and blue inner ring represents ASD subjects belonging to subtype 2. The outer circle indicates the network corresponding to the mean IDFC. **D** Mean IDFC patterns at the region level of two ASD subtypes. The two inner rings represent the mean IDFC of ROIs, and the outer ring indicates the network corresponding to ROIs. Red inner ring represents ASD subjects belonging to subtype 1, and blue inner ring represents ASD subjects belonging to subtype 2. IDFC, inter-individual deviation of functional connectivity; ASD, autism spectrum disorder; ROI, region of interest; SMN, somatosensory network; CO, cingulo-opercular network; AN, auditory network; DMN, default-mode network; VN, visual network; FPN, fronto-parietal network; SAN, salience network; SUB, subcortical network; VAN, ventral attention network; DAN, dorsal attention network; and UND, uncertain network

The optimal value of k for the k-means clustering was determined as 2, since it can be seen from Fig. 2A that the maximum value of the mean silhouette is 0.61 at *k* = 2. The 105 ASDs were classified into two ASD subtypes (47 subjects in subtype 1 and 58 subjects in subtype 2) by *k*-means clustering based on IDFC at the network level, as shown in Fig. 2B. The IDFC patterns of the two ASD subtypes at the network level and at the brain region level are shown in Fig. 2C, D, and the IDFC patterns are also presented in table form as detailed in Additional file 1: Tables S1 and S2.

No significant differences were found between ASD subtype 1 and ASD subtype 2 in terms of demographics, including: age, FIQ, handedness, eye status, and mean FD. And no significant differences were found between the two ASD subtype groups in terms of clinical symptom severity by comparing ADOS scores either. Considering that we used multicenter data of subjects, we also examined the data center distribution of both ASD subtypes (Additional file 1: Fig. S1; see Additional file 1 for details).

### Atypical FC patterns of ASD subtypes

**Fig. 3:**
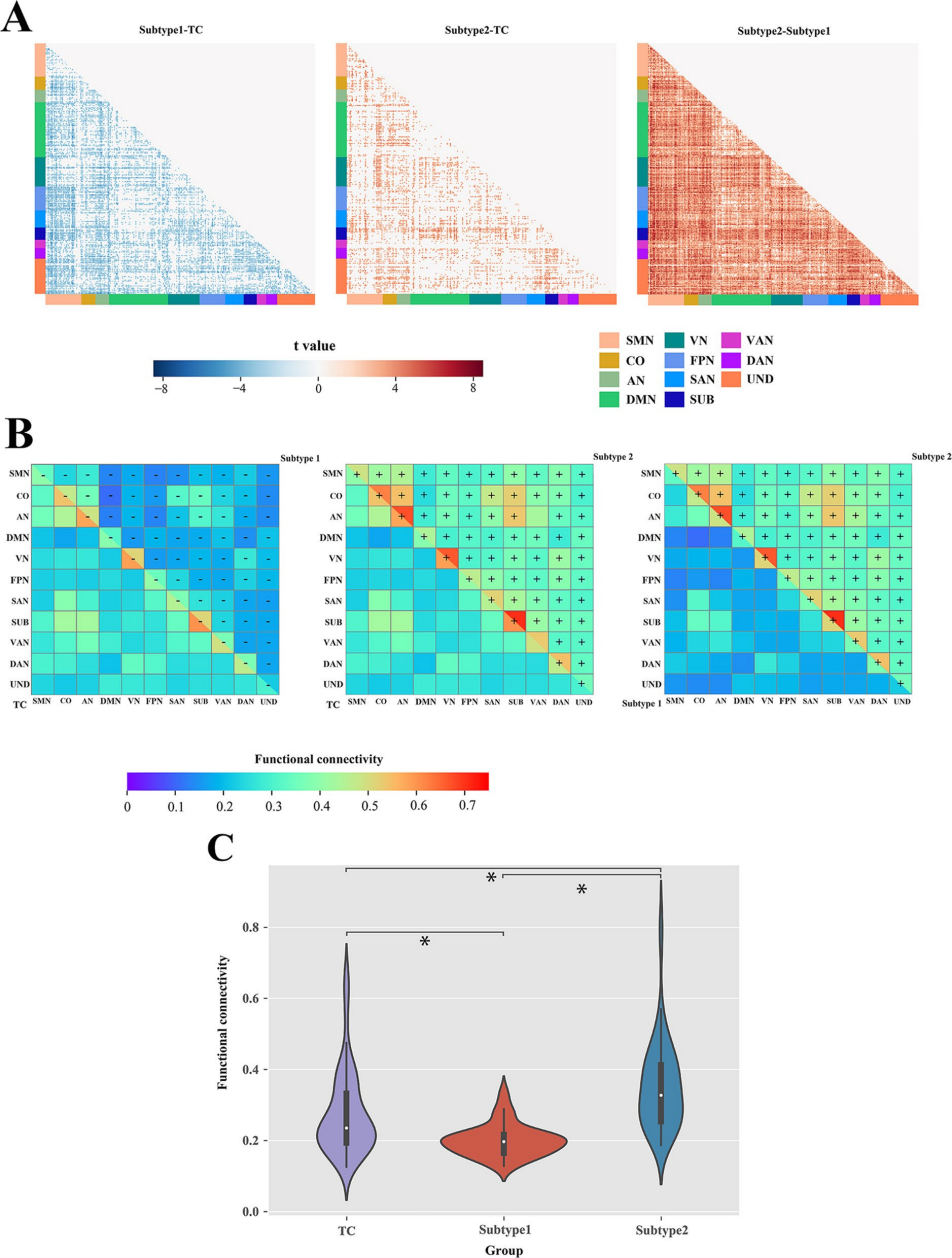
Analysis of atypical FC patterns in ASD subtypes. **A** Results of two-sample t tests for significant differences in FC at the region level for subtype 1, subtype 2, and TC (*p* < 0.05, FDR corrected). **B** Differences in FC of subtype 1, subtype 2, and TC at the network level. The upper right and lower left triangles indicate the average network FC values for the different groups. ± indicates a significant increase or decrease in FC for the group in the upper right compared to the group in the lower left (two-sample t tests, *p* < 0.05, FDR corrected). **C** Differences in FC of subtype 1, subtype 2, and TC at the whole-brain level. *represents significant differences (two-sample t tests, *p* < 0.05, Bonferroni corrected). FC, Functional connectivity; ASD, autism spectrum disorder; FDR, false discovery rate; TC, typical controls; SMN, somatosensory network; CO, cingulo-opercular network; AN, auditory network; DMN, default-mode network; VN, visual network; FPN, fronto-parietal network; SAN, salience network; SUB, subcortical network; VAN, ventral attention network; DAN, dorsal attention network; and UND, uncertain network

FC comparison analysis at the region, network, and whole-brain levels was performed for subtype 1, subtype 2, and TC groups, using two-sample t tests, as shown in Fig. 3. Of the 34,716 possible connectivity edges at the region level, the aberrant FC pattern was found to be significantly lower for the subtype 1 group than for the TC group on 37% of the edges (i.e., 12,700 edges), significantly higher for the subtype 2 group than for the TC group on 16% of the edges (i.e., 5623 edges), and significantly higher for the subtype 2 group than for the subtype 1 group on 90% of the edges (i.e., 31,388 edges) (*p* < 0.05, FDR corrected) (Fig. 3A). In the comparison of the whole ASD and TC groups, no significantly different connectivity edges were found, as detailed in Additional file 1: Fig. S2.

Significant differences were also found in the network-level FC analysis (including intra-network connectivity and inter-network connectivity). The subtype 1 group showed a significant decrease in FC on all 66 connectivity edges compared to the TC group, the subtype 2 group showed a significant increase in FC on 64 connectivity edges compared to the TC group, and the subtype 2 group showed a significant increase in FC on all 66 connectivity edges compared to the subtype 1 group (*p* < 0.05, FDR corrected) (Fig. 3B). Analysis of the t-values of the two-sample t tests for network-level FC difference analysis revealed that the absolute value of t-values was larger between ASD subtype 1 and TC in SMN-FPN, SMN-SUB, and VAN-VAN, and between ASD subtype 2 and TC in SUB-DMN, SUB-VN, SUB-FPN, and SUB-DAN, as detailed in Additional file 1: Fig. S4A. The analysis of the percentage of connectivity edges with significant FC differences in ROI between networks showed that the percentage of connectivity edges with significant FC differences in ROI between ASD subtype 1 and TC was larger at SMN-FPN, SMN-SUB, CO-DAN, AN-FPN, AN-SUB, and VAN-VAN, and the percentage of connectivity edges with significant FC differences in ROI between ASD subtype 2 and TC was larger at SUB-DMN, SUB-VN, SUB-FPN, and SUB-DAN, as detailed in Additional file 1: Fig. S4B. In the comparison of the whole ASD and TC groups, no significantly different network connectivity edges were found, as detailed in Additional file 1: Fig. S5.

Finally, the FC at the whole-brain level was analyzed and the subtype 1 group showed a significant decrease in FC compared to the TC group, the subtype 2 group showed a significant increase in FC compared to the TC group, and the subtype 2 group showed a significant increase in FC compared to the subtype 1 group (*p* < 0.05, Bonferroni corrected) (Fig. 3C). In contrast, no significant differences were found comparing the whole ASD group with the TC group, as detailed in Additional file 1: Fig. S6.

### Relationship between IDFC of ASD subtypes and severity of ASD clinical symptoms

**Fig. 4:**
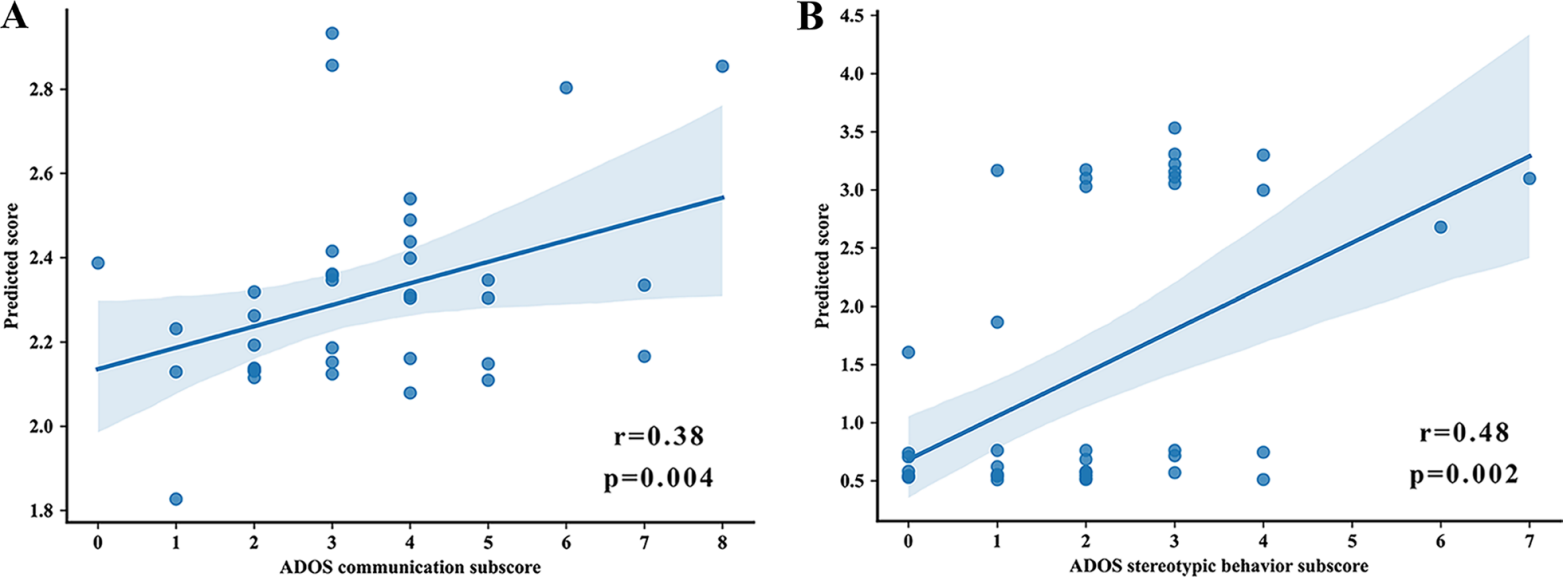
Relationships between ADOS subscores and predicted scores using network-level IDFC. **A** The relationship between ADOS communication subscore and predicted scores for ASD subtype 1. **B** The relationship between ADOS stereotypic behavior subscore and predicted scores for ASD subtype 2. ADOS, Autism Diagnostic Observation Schedule; IDFC, inter-individual deviation of functional connectivity; and ASD, autism spectrum disorder

For each ASD subtype, the multivariate support vector regression model was used to investigate the relationship between IDFC and ASD symptom severity. Model performance was further assessed using LOOCV, and statistical significance was determined by a non-parametric permutation test. The network-level IDFC of ASD subtype 1 predicted the ADOS communication subscore (*r* = 0.38, *p* = 0.004; Fig. 4A), and no significant relationship was observed with other subscores of ADOS. The network-level IDFC of ASD subtype 2 predicted the ADOS stereotypic behavior subscore (*r* = 0.48, *p* = 0.002; Fig. 4B), and no significant relationship was observed with other subscores of ADOS. The feature weight analysis resulted in ASD subtype 1 exhibiting higher weights for VN, VAN, and DAN and lower weights for DMN, FPN, and SUB, and ASD subtype 2 exhibiting higher weights for SMN, VN, and DAN and lower weights for CO and SUB, as shown in Additional file 1: Fig. S7.

### Within-group inter-individual FC deviation differences between the ASD and TC groups

**Fig. 5:**
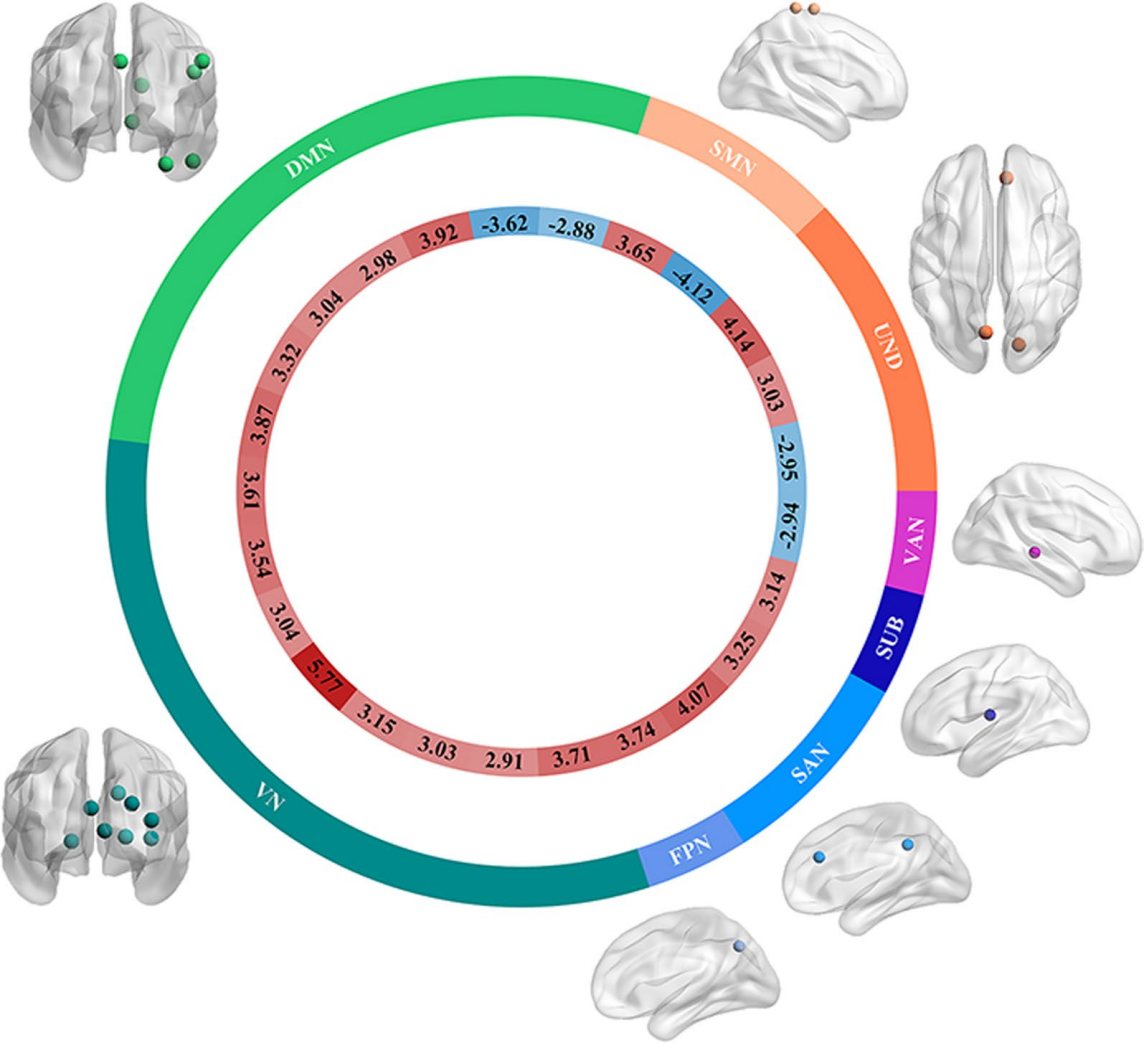
Results of two-sample t tests for within-group inter-individual FC deviation at the region level of ASD and TC groups. The brain map shows the location of the ROIs (with significant differences between ASD and TC). The outer circle indicates the network where the ROIs are located. The inner circle indicates the t-value of the two-sample t tests (*p* < 0.05, FDR corrected). FC, Functional connectivity; ASD, autism spectrum disorder; TC: typical controls; ROI, region of interest; FDR, false discovery rate; SMN, somatosensory network; DMN, default-mode network; VN, visual network; FPN, fronto-parietal network; SAN, salience network; SUB, subcortical network; VAN, ventral attention network; and UND, uncertain network

The differences in within-group inter-individual FC deviation between the ASD and TC groups were analyzed from the ROI level using two-sample t tests. Compared with the TC group, the ASD group showed higher within-group inter-individual FC deviation at 20 ROIs and lower at 5 ROIs (*p* < 0.05, FDR corrected), as shown in Fig. 5. Among them, the 20 ROIs with higher within-group inter-individual FC deviation include: one in SMN, five in DMN, eight in VN, one in FPN, two in SAN, one in SUB, and two in UND. The 5 ROIs with lower within-group inter-individual FC deviation included one in SMN, two in DMN, one in VAN, and one in UND. This result is also presented in table form as detailed in Additional file 1: Table S3.

## Discussion

In this study, IDFC constructed based on FC networks was used to analyze the heterogeneity of functional brain deviations in children with ASD. Two ASD subtypes were obtained by IDFC-based ASD clustering analysis, and aberrant FC patterns of ASD subtypes were identified. The IDFC of ASD subtypes was found to predict the severity of different ASD symptoms separately by a multivariate support vector regression model. By comparing the within-group inter-individual FC deviation of the ASD group with that of the TC group, a complex pattern of differences in brain functional network heterogeneity was found. These results further illustrate the heterogeneity of ASD from the perspective of functional brain networks and provide a new way to study brain heterogeneity in individuals with ASD.

### Subtypes of individuals with ASD

ASD can be divided into different subtypes in terms of brain structure [29] and brain function [20], as reported in previous studies. Subtyping is widely used in many aspects of ASD research. While 25–40% of children with ASD showed normal brain development in early life and were found to have symptoms of ASD after 18 months of life, there were also children with ASD who were found to have developmental delays within 18 months, and this possibly suggests that there were different subtypes of ASD at the level of brain development [41]. In a study of subtypes of ASD, two subtypes of ASD with differential FC patterns in different resting-state networks were identified, and the two subtypes differed in clinical symptom severity [42]. Another subtype study based on FC showed that each ASD subtype had unique behavioral brain relationships, suggesting the possibility of clinical applications for differentiating ASD subtypes based on FC [29]. Consistent with previous studies, we also support that ASD can be subdivided into subtypes, and the present study reveals two ASD subtypes with different IDFC patterns, further revealing a heterogeneous pattern of functional brain networks in ASD from the perspective of inter-individual deviation.

Although heterogeneity in ASD has been explained by subtypes from different perspectives in previous studies, studies have also attributed heterogeneity in ASD to factors such as age [43–45], intelligence [46], and gender [47]. To exclude the effects of these factors, only male subjects were used in this study and age and FIQ were regressed out prior to subtype identification, and two highly heterogeneous ASD subtypes were finally obtained. That is, the difference in IDFC between the two ASD subtypes was not influenced by age, intelligence, and gender. This means that the results of the present study are not due to these factors, but more likely to the inherent heterogeneity of the functional network of the ASD brain, which reinforces the need for subtyping in the future studies of ASD.

### Atypical FC pattern of ASD subtypes

Recent ASD subtype studies reported that different ASD subtypes have different atypical FC patterns [29, 48], and we found similar results for ASD subtype studies based on IDFC. We obtained ASD subtype 1 exhibiting a lower functional connectivity pattern, especially among SMN-FPN, SMN-SUB, and VAN-VAN networks, and ASD subtype 2 exhibiting a higher functional connectivity pattern, especially among SUB-DMN, SUB-VN, SUB-FPN, and SUB-DAN networks, which is similar to the previous findings. Previous studies on ASD brain function reported anomalous FC patterns between SUB network and other networks [49], and anomalous FC between SMN and FPN network was also reported [20]. The IDFC of the two ASD subtypes predicted different ASD symptom severities possibly attributed to the different atypical FC patterns of the two subtypes, which further illustrates the important role of the two ASD subtypes obtained in this study.

In this study, a subtyping approach was used to study FC abnormality patterns, and IDFC was used as a subtyping feature to emphasize the influence of brain heterogeneity on ASD more than traditional features. The two ASD subtype groups exhibited hypoconnectivity pattern and hyperconnectivity pattern relative to the TC group, and in line with our conjecture, no significant differences were found when comparing the FC of the whole ASD group with that of the TC group. Previous studies have identified complex patterns of FC in resting-state functional brain network studies in ASD, and inconsistent results are common [15]. Studying the entire ASD group led to some potential atypical FC patterns being overlooked or misinterpreted, which is an important reason why the results of previous studies are often inconsistent.

### Relationships between IDFC and severity of ASD clinical symptoms

Previous studies have generally reported that abnormal functional networks potentially related to clinical symptoms in individuals with ASD [23, 50]. Clinical correlation analysis based on ASD subtypes to obtain a better prediction of ADOS scores has also been reported [19], which suggests a link between the heterogeneity of brain networks in individuals with ASD and the heterogeneity of their clinical symptom severity. This view is also supported by our study, in which both ASD subtypes of IDFC were correlated with ASD symptoms but did not behave in the same way, with subtype 1 showing a potential association with the severity of social communication impairments in ASD and subtype 2 showing a relationship with ASD symptoms in terms of severity of restricted and repetitive behaviors. Previous studies also found different potential relationships between different ASD subtypes and ASD symptoms. A previous study classified ASD into three subtypes: Asperger’s, Pervasive Developmental Disorder-Not otherwise Specified, and Autism, and each subtype-specific brain pattern is correlated with different ADOS subdomains [51]. The correlation of one ASD subtype with core ASD symptoms was reported in a subtype study of ASD by resting-state FC and was not found in other subtypes [52]. ASD subtyping studies hold promise as a way to reflect the clinical heterogeneity of ASD.

The two ASD subtypes showed different network feature weights in the brain–behavioral analysis, indicating that the contribution of different functional networks to the prediction of symptoms differed between the two subtypes, and this difference could potentially account for the difference between the relationships between the two subtypes and ASD symptoms; this difference in relationship could also be attributed to the hypoconnectivity and hyperconnectivity patterns of the two subtypes. Our study on the potential relationship between deviant features of brain networks and clinical symptoms may provide a promising way to explain the high heterogeneity of clinical symptoms of ASD and boost the development of clinical diagnostic markers.

### Inter-individual FC deviation within the group differences between the ASD and TC groups

Brain heterogeneity in ASD is different from that in TC in the present study. The ASD group showed higher inter-individual FC deviation within the group at 20 ROIs and lower levels at 5 ROIs, i.e., greater individual differences in FC at more ROIs. The results of the restudy using two TC subgroups also found greater individual differences in FC at more ROIs for ASD compared to the TC subgroups. This suggests broader inter-individual variation among ASD compared to TC, consistent with previous findings on individuals brain heterogeneity in ASD [16, 21, 29]. Moreover, significantly different ROIs were located in multiple networks (including 7 functional networks and an uncertain network), and significantly different ROIs were also found to be located in multiple networks in a complementary analysis using two TC subgroups, which further corroborates the high complexity of ASD and the need to study it on a large scale [53, 54].

## Limitations

First, due to the relatively limited number of female participants in the ABIDE database, only male children were selected, which prevented analysis of the effects of gender. Because gender is a factor that influences ASD heterogeneity [55], there is a need to focus on collecting data on female individuals with ASD in future studies to be able to explore gender heterogeneity in ASD. Second, previous studies have generally concluded that ASD is a developmental disorder [56, 57] and the heterogeneity of functional brain networks of individuals with ASD at other developmental stages, such as adolescence and adulthood, remains unknown. In order to figure out the development of heterogeneity of functional brain networks in ASD, longitudinal data including subjects at different developmental stages are needed for future longitudinal studies. Third, recent studies have shown that resting-state FC is a dynamic process [31, 58, 59], and it is necessary to develop dynamic subtyping methods in the future, which may better reveal ASD heterogeneity. Finally, the present study was a unimodal study based on inter-individual heterogeneity of functional brain networks and the influence of structural brain networks needs to be considered in future studies. A multimodal study that introduces structural brain networks could provide a more comprehensive analysis of heterogeneity in ASD.

## Conclusion

This study focused on the heterogeneity among individuals with ASD based on functional brain networks deviations of ASD from TC, revealing two ASD subtypes with distinct FC patterns at multiple spatial scales. Furthermore, IDFC of ASD subtypes was found to predict the severity of different ASD symptoms. These results reveal individual heterogeneity of functional brain networks in ASD and highlight the important role of individual heterogeneity in explaining the complex brain patterns of ASD.

## Supplementary Information

**Additional file 1**. **Supplementary materials**, fig. S1-S11, and tables S1-S6.

## Data Availability

The data that support the findings of this study are available in Autism Brain Imaging Data Exchange (ABIDE, fcon_1000.projects.nitrc.org/indi/abide/) database.

## Abbreviations

ASD: Autism spectrum disorder
TC: Typical controls
FC: Functional connectivity
IDFC: Inter-individual deviation of functional connectivity
ADOS: Autism Diagnostic Observation Schedule
ADOS_COMM: Communication subscore of the ADOS
ADOS_SOCIAL: Social subscore of the ADOS
ADOS_RRB: Stereotyped behaviors and restricted interests subscore of the ADOS
fMRI: Functional magnetic resonance imaging
ABIDE: Autism Brain Imaging Data Exchange database
FD: Frame-wise displacement
FIQ: Full intelligence quotient
DPARSF: Data Processing Assistant for Resting-State fMRI
MNI: Montreal Neurological Institute
ROI: Region of interest
SMN: Somatosensory network
CO: Cingulo-opercular network
AN: Auditory network
DMN: Default-mode network
VN: Visual network
FPN: Fronto-parietal network
SAN: Salience network
SUB: Subcortical network
VAN: Ventral attention network
DAN: Dorsal attention network
UND: Uncertain network
FDR: False discovery rate
LIBLINEAR: Library for Large Linear Classification
LOOCV: Leave-one-out cross-validation
