# Supplementary material for: Inter-individual heterogeneity of functional brain networks in children with autism spectrum disorder
Source: Mol Autism. 2022 Dec 26;13:52. doi: 10.1186/s13229-022-00535-0 (PMC9793594; doi:10.1186/s13229-022-00535-0)
Supplement: Supplementary file 1 — Additional file 1. Supplementary materials, fig. S1-S11, and tables S1-S6. [file 13229_2022_535_MOESM1_ESM.docx]

**Supplementary materials**

**Inter-individual heterogeneity of functional brain networks in children with autism spectrum disorder**

Xiaonan Guo^1, 2*^, Guangjin Zhai^1, 2^, Junfeng Liu^3^, Yabo Cao^1, 2^, Xia Zhang^1, 2^, Dong Cui^1, 2^, Le Gao^1, 2*^

1. School of Information Science and Engineering, Yanshan University, Qinhuangdao, 066004, China
2. Hebei Key Laboratory of Information Transmission and Signal Processing, Yanshan University, Qinhuangdao, 066004, China
3. Department of Neurology, West China Hospital, Sichuan University, Chengdu, 610041, China

* Corresponding author: Xiaonan Guo and Le Gao. School of Information Science and Engineering, Yanshan University, Qinhuangdao, 066004, China

E-mails: guoxiaonan@ysu.edu.cn (Xiaonan Guo), gaole@ysu.edu.cn (Le Gao)

**Results**

1. **Data center distribution of ASD subtypes**

The data center distribution of the two ASD subtypes was examined and clustering results were not influenced by the source of the subjects (Figure S1).


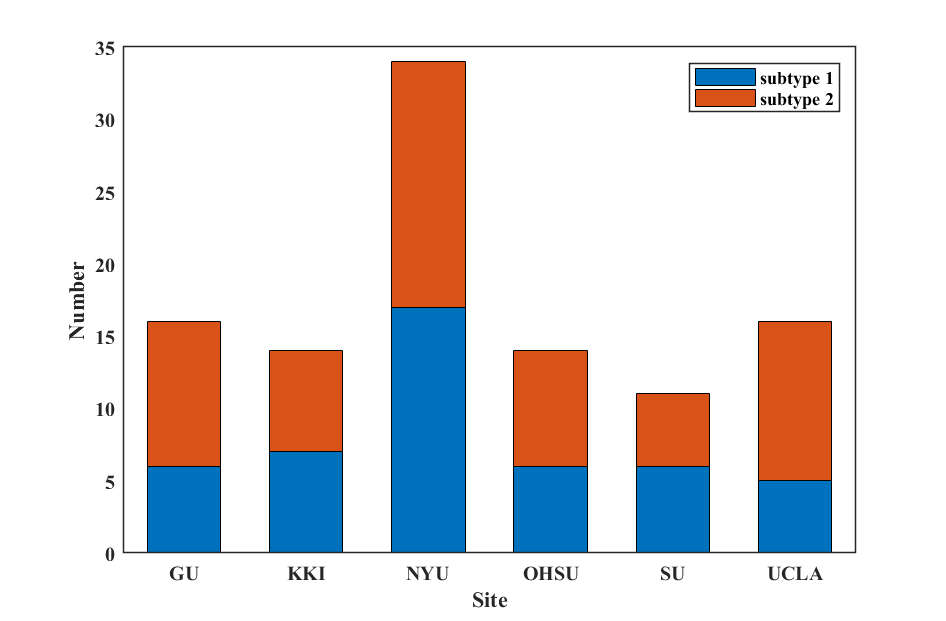


**Figure S1. Data center distribution of the two ASD subtypes.** GU, Georgetown University; KKI, Kennedy Krieger Institute; NYU, New York University Langone; OHSU, Oregon Health and Science University; SU, Stanford University; UCLA, University of California Los Angeles.

1. **Analysis of FC differences between the whole ASD group and the TC group at the region level**

FC differences between the whole ASD and TC groups at the region level were analyzed by two-sample t-tests (p < 0.05, FDR corrected), and no significantly different connectivity edges were found (Figure S2).

**
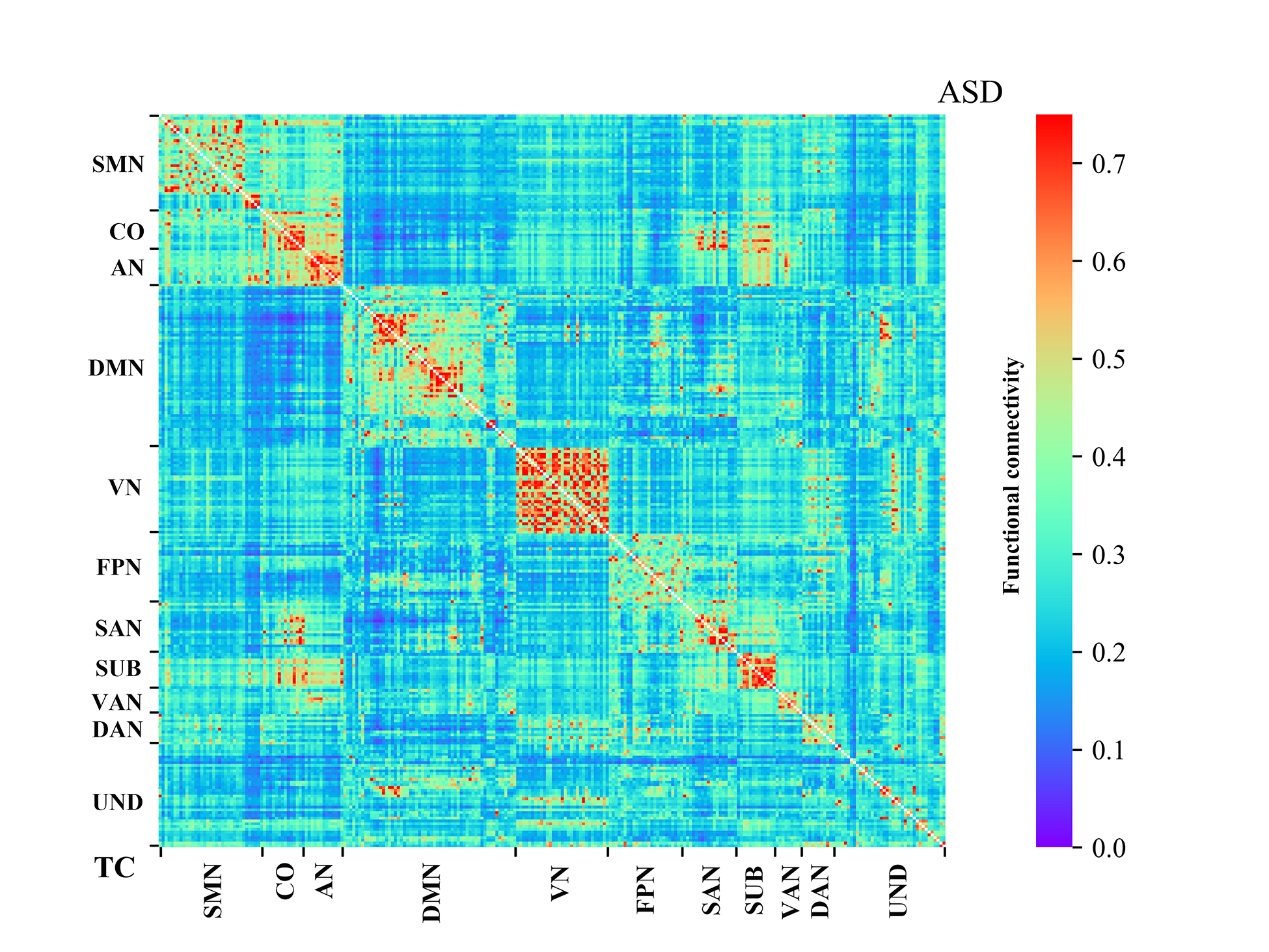
**

**Figure S2.** **Results of the FC comparison between the whole ASD group and the TC group at the region level.** SMN, somatosensory network; CO, cingulo-opercular network; AN, auditory network; DMN, default-mode network; VN, visual network; FPN, fronto-parietal network; SAN, salience network; SUB, subcortical network; VAN, ventral attention network; DAN, dorsal attention network; UND, uncertain network.

1. **Analysis of FC differences between the ASD subtypes and the TC** **subgroups at the network level**

Network-level IDFC were calculated using the ASD group and TC subgroup 1 (51 subjects) and performed to cluster analysis, and then two ASD subtypes (47 subjects in subtype 1 and 58 subjects in subtype 2) were obtained. The same analysis was performed using the ASD group and TC subgroup 2 (51 subjects), and two ASD subtypes (47 subjects in subtype 1 and 58 subjects in subtype 2) were obtained. We compared the network-level FC between the two ASD subtypes and TC subgroup 1, and the network-level FC between the two ASD subtypes and TC subgroup 2 was also compared. The analysis process is the same as when using the whole TC group for analysis. Significant differences were found in the network-level FC analysis (including intra-network connectivity and inter-network connectivity). Compared to the TC subgroup 1, the ASD subtype 1 group (47 individuals with ASD) showed a significant decrease in FC on all 66 connectivity edges, the ASD subtype 2 group (58 individuals with ASD) showed a significant increase in FC on 55 connectivity edges. In addition, the ASD subtype 2 group showed a significant increase in FC on all 66 connectivity edges compared to the ASD subtype 1 group (p < 0.05, FDR corrected) (Figure S3A). Compared to the TC subgroup 2, the ASD subtype 1 group (47 individuals with ASD) showed a significant decrease in FC on all 66 connectivity edges, the ASD subtype 2 group (58 individuals with ASD) showed a significant increase in FC on 53 connectivity edges. In addition, the ASD subtype 2 group showed a significant increase in FC on all 66 connectivity edges compared to the ASD subtype 1 group (p < 0.05, FDR corrected) (Figure S3B).

**
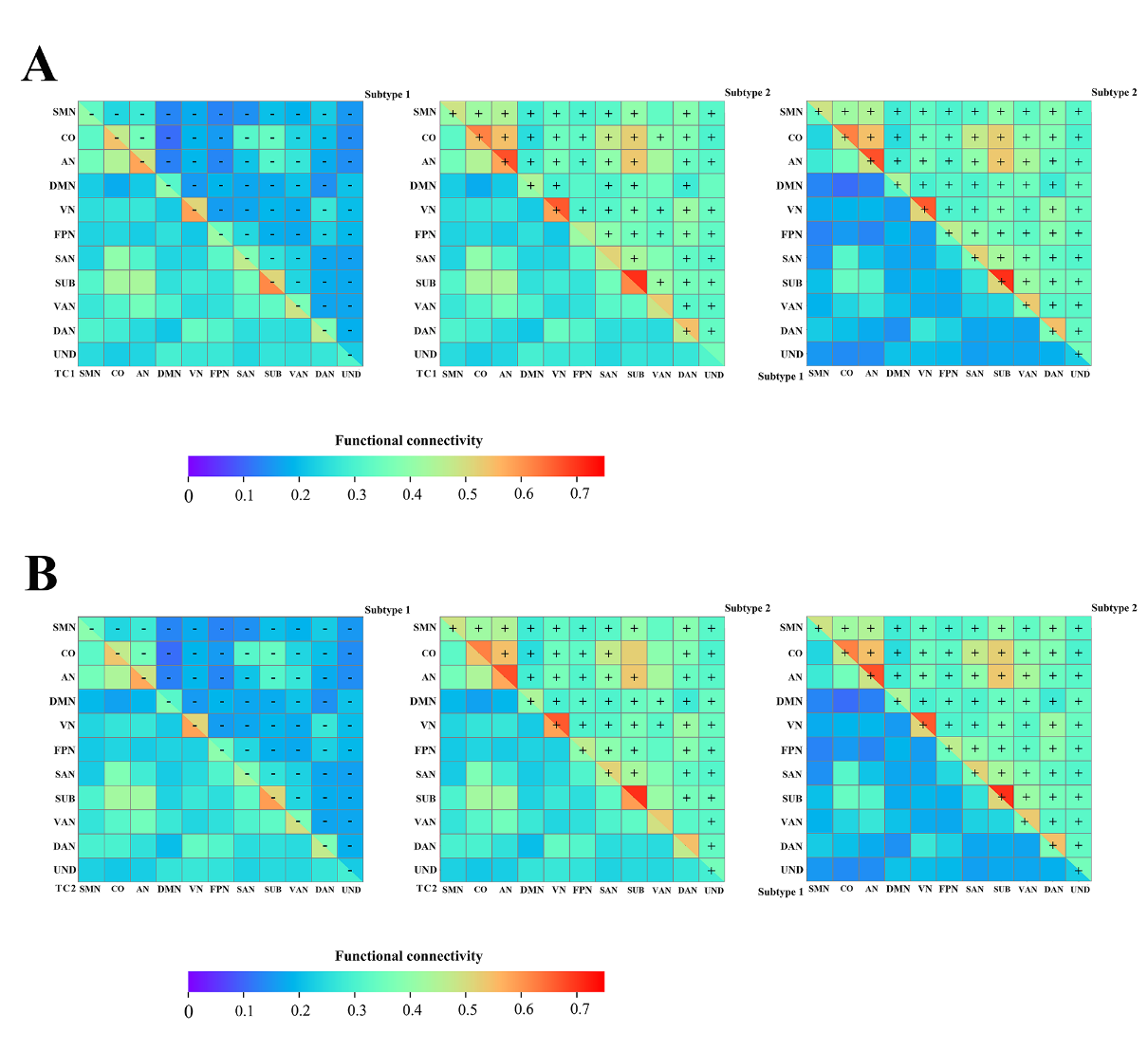
**

**Figure S3. Results of FC difference analysis at the network level between ASD subtypes and TC subgroups.** The upper right and lower left triangles indicate the average network FC values for the different groups. +/- indicates a significant increase or decrease in FC for the group in the upper right compared to the group in the lower left (two-sample t-tests, p < 0.05, FDR corrected). SMN, somatosensory network; CO, cingulo-opercular network; AN, auditory network; DMN, default-mode network; VN, visual network; FPN, fronto-parietal network; SAN, salience network; SUB, subcortical network; VAN, ventral attention network; DAN, dorsal attention network; UND, uncertain network.

1. **Atypical FC patterns at the network level for the ASD subtypes**

The t-values of the two-sample t-tests for network-level FC difference analysis are shown in Figure S4A, where the absolute value of t-values between ASD subtype 1 and TC are larger at SMN-FPN, SMN-SUB and VAN-VAN, and the absolute value of t-values between ASD subtype 2 and TC are larger at SUB-DMN, SUB-VN, SUB-FPN and SUB-DAN. The percentage of connectivity edges with significant FC differences in ROI between networks is shown in Figure S4B, where the percentage of connectivity edges with significant FC differences in ROI between ASD subtype 1 and TC is larger at SMN-FPN, SMN-SUB, CO-DAN, AN-FPN, AN-SUB, and VAN-VAN, and the percentage of connectivity edges with significant FC differences in ROI between ASD subtype 2 and TC is larger at SUB-DMN, SUB-VN, SUB-FPN and SUB-DAN.


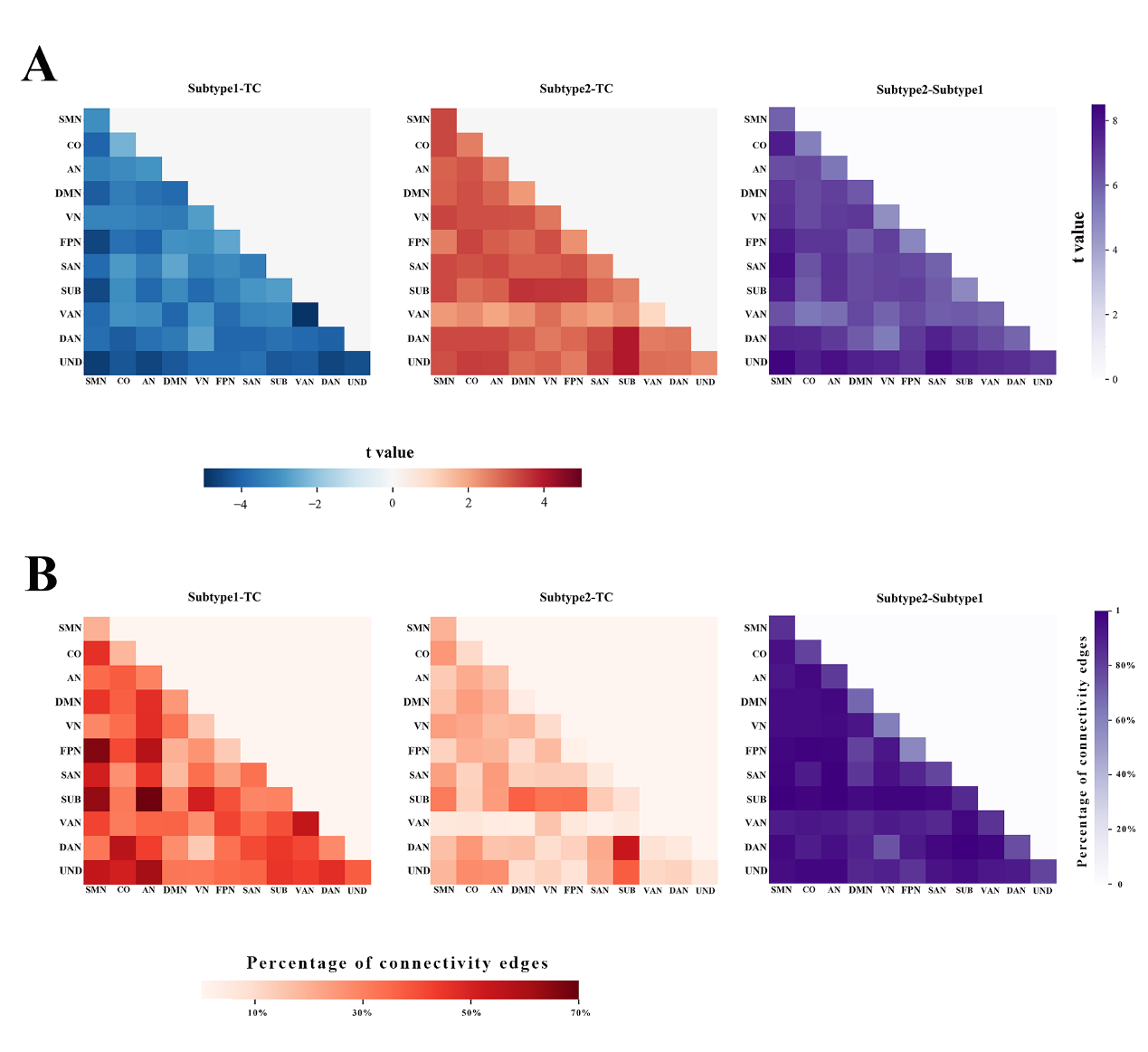


**Figure S4. Results of network-level FC difference analysis of ASD subtypes.** (A) The results of the two-sample t-tests for network-level FC difference analysis. (B) The percentage of connectivity edges with significant FC differences in ROI between networks. SMN, somatosensory network; CO, cingulo-opercular network; AN, auditory network; DMN, default-mode network; VN, visual network; FPN, fronto-parietal network; SAN, salience network; SUB, subcortical network; VAN, ventral attention network; DAN, dorsal attention network; UND, uncertain network.

1. **Analysis of FC differences between the whole ASD group and the TC group at the** **network level**

FC differences between the whole ASD and TC groups at the network level were analyzed by two-sample t-tests (p < 0.05, FDR corrected), and no significantly different network connectivity edges were found (Figure S5).


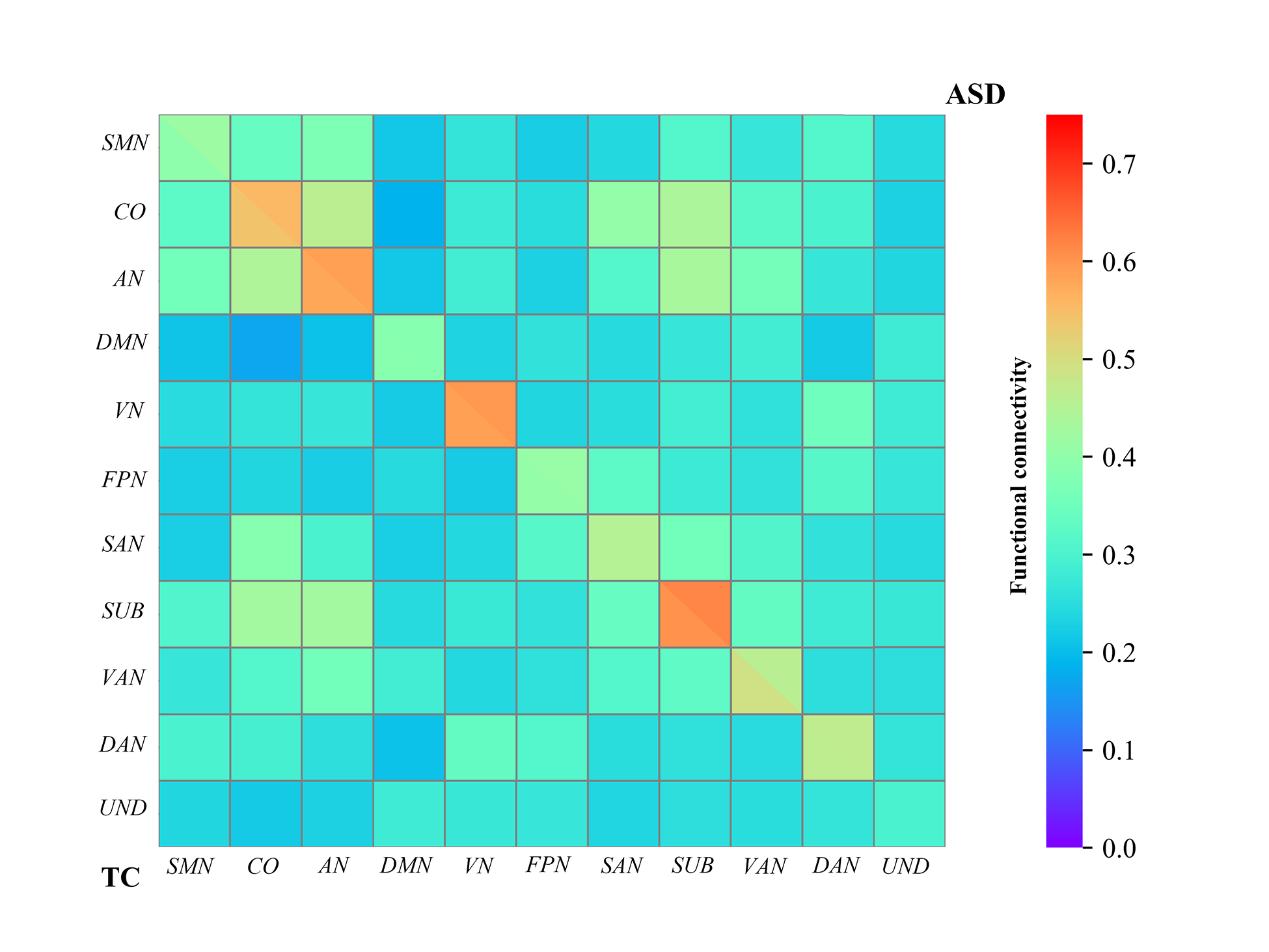


**Figure S5.** **Results of the FC comparison between the whole ASD group and the TC group at the network level.** SMN, somatosensory network; CO, cingulo-opercular network; AN, auditory network; DMN, default-mode network; VN, visual network; FPN, fronto-parietal network; SAN, salience network; SUB, subcortical network; VAN, ventral attention network; DAN, dorsal attention network; UND, uncertain network.

1. **Analysis of FC differences between the whole ASD group and the TC group at the whole-brain level**

FC differences between the whole ASD and TC groups at the whole-brain level were analyzed by two-sample t-tests, and no significantly differences were found (Figure S6).


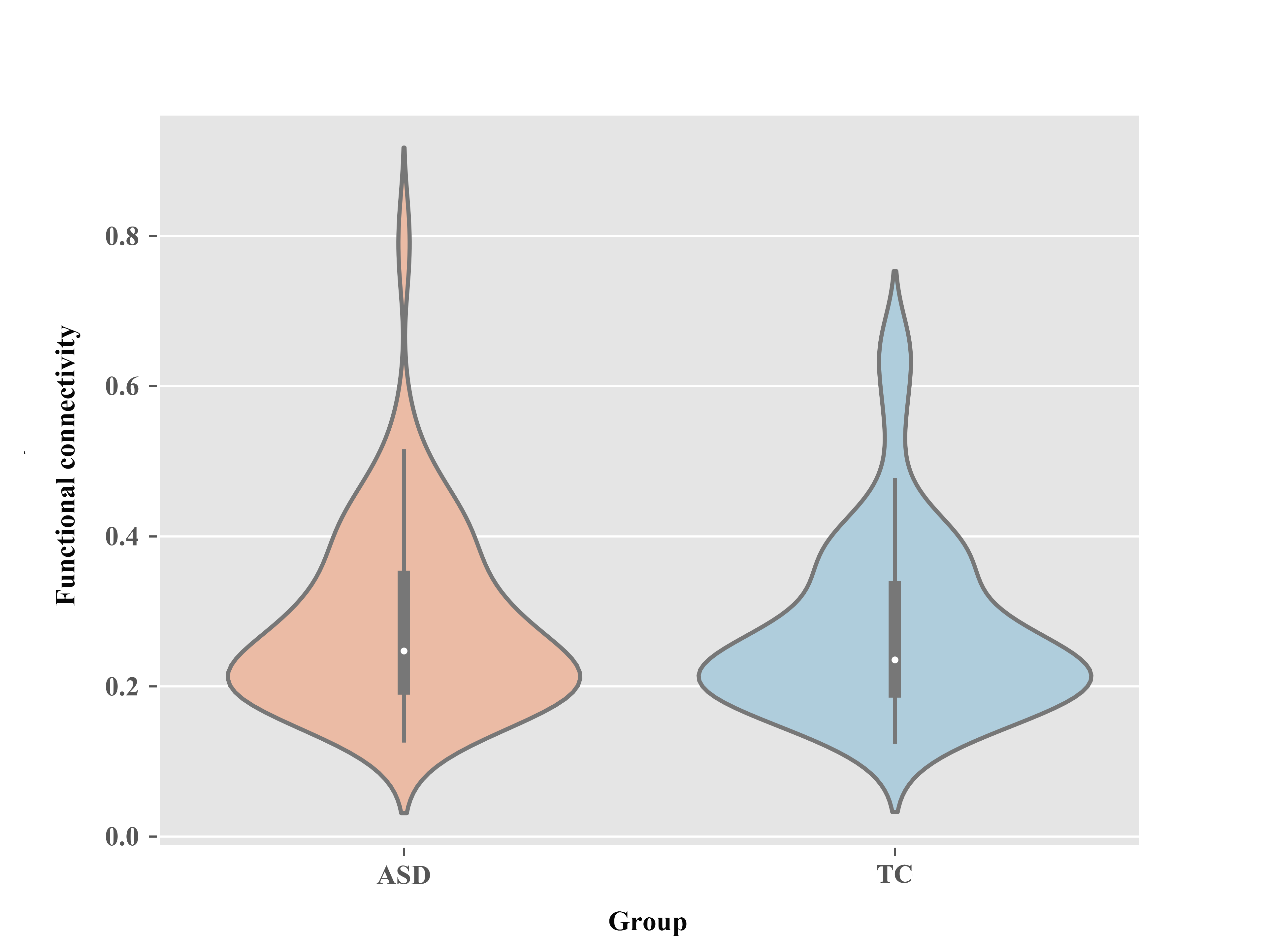


**Figure S6. Results of the FC comparison between the whole ASD group and the TC group at the whole-brain level.**

1. **Mean IDFC patterns of two ASD subtypes**

**Table S1. Mean IDFC patterns at the network level of two ASD subtypes.**

|  | SMN | CO | AN | DMN | VN | FPN | SAN | SUB | VAN | DAN | UND |
| --- | --- | --- | --- | --- | --- | --- | --- | --- | --- | --- | --- |
| Mean IDFC |  |  |  |  |  |  |  |  |  |  |  |
| subtype 1 | 0.52 | 0.49 | 0.48 | 0.48 | 0.45 | 0.56 | 0.51 | 0.49 | 0.52 | 0.59 | 0.59 |
| subtype 2 | 0.46 | 0.44 | 0.42 | 0.43 | 0.41 | 0.50 | 0.45 | 0.43 | 0.46 | 0.53 | 0.53 |

SMN, somatosensory network; CO, cingulo-opercular network; AN, auditory network; DMN, default-mode network; VN, visual network; FPN, fronto-parietal network; SAN, salience network; SUB, subcortical network; VAN, ventral attention network; DAN, dorsal attention network; UND, uncertain network.

**Table S2. Mean IDFC patterns at the region level of two ASD subtypes.**

| Brain networks | Nodes | MNI coordinates | | | Mean IDFC | |
| --- | --- | --- | --- | --- | --- | --- |
|  |  | *x* | *y* | *z* | subtype 1 | subtype 2 |
| SMN |  |  |  |  |  |  |
|  | 1 | -7 | -52 | 61 | 0.52 | 0.45 |
|  | 2 | -14 | -18 | 40 | 0.65 | 0.60 |
|  | 3 | 0 | -15 | 47 | 0.49 | 0.42 |
|  | 4 | 10 | -2 | 45 | 0.43 | 0.37 |
|  | 5 | -7 | -21 | 65 | 0.48 | 0.41 |
|  | 6 | -7 | -33 | 72 | 0.46 | 0.38 |
|  | 7 | 13 | -33 | 75 | 0.52 | 0.43 |
|  | 8 | -54 | -23 | 43 | 0.60 | 0.55 |
|  | 9 | 29 | -17 | 71 | 0.57 | 0.50 |
|  | 10 | 10 | -46 | 73 | 0.57 | 0.50 |
|  | 11 | -23 | -30 | 72 | 0.44 | 0.37 |
|  | 12 | -40 | -19 | 54 | 0.56 | 0.50 |
|  | 13 | 29 | -39 | 59 | 0.58 | 0.52 |
|  | 14 | 50 | -20 | 42 | 0.57 | 0.51 |
|  | 15 | -38 | -27 | 69 | 0.50 | 0.42 |
|  | 16 | 20 | -29 | 60 | 0.53 | 0.46 |
|  | 17 | 44 | -8 | 57 | 0.48 | 0.41 |
|  | 18 | -29 | -43 | 61 | 0.54 | 0.48 |
|  | 19 | 10 | -17 | 74 | 0.38 | 0.32 |
|  | 20 | 22 | -42 | 69 | 0.56 | 0.51 |
|  | 21 | -45 | -32 | 47 | 0.63 | 0.57 |
|  | 22 | -21 | -31 | 61 | 0.54 | 0.48 |
|  | 23 | -13 | -17 | 75 | 0.55 | 0.51 |
|  | 24 | 42 | -20 | 55 | 0.48 | 0.41 |
|  | 25 | -38 | -15 | 69 | 0.59 | 0.52 |
|  | 26 | -16 | -46 | 73 | 0.54 | 0.47 |
|  | 27 | 2 | -28 | 60 | 0.49 | 0.42 |
|  | 28 | 3 | -17 | 58 | 0.46 | 0.40 |
|  | 29 | 38 | -17 | 45 | 0.54 | 0.48 |
|  | 30 | -49 | -11 | 35 | 0.43 | 0.38 |
|  | 31 | 36 | -9 | 14 | 0.48 | 0.41 |
|  | 32 | 51 | -6 | 32 | 0.48 | 0.43 |
|  | 33 | -53 | -10 | 24 | 0.45 | 0.40 |
|  | 34 | 66 | -8 | 25 | 0.49 | 0.44 |
|  | 35 | 47 | -30 | 49 | 0.57 | 0.51 |
| CO |  |  |  |  |  |  |
|  | 1 | -3 | 2 | 53 | 0.43 | 0.37 |
|  | 2 | 54 | -28 | 34 | 0.55 | 0.50 |
|  | 3 | 19 | -8 | 64 | 0.58 | 0.54 |
|  | 4 | -16 | -5 | 71 | 0.65 | 0.60 |
|  | 5 | -10 | -2 | 42 | 0.52 | 0.46 |
|  | 6 | 37 | 1 | -4 | 0.44 | 0.39 |
|  | 7 | 13 | -1 | 70 | 0.56 | 0.50 |
|  | 8 | 7 | 8 | 51 | 0.40 | 0.35 |
|  | 9 | -45 | 0 | 9 | 0.46 | 0.41 |
|  | 10 | 49 | 8 | -1 | 0.41 | 0.37 |
|  | 11 | -34 | 3 | 4 | 0.43 | 0.38 |
|  | 12 | -51 | 8 | -2 | 0.46 | 0.40 |
|  | 13 | -5 | 18 | 34 | 0.43 | 0.38 |
|  | 14 | 36 | 10 | 1 | 0.50 | 0.46 |
| AN |  |  |  |  |  |  |
|  | 1 | 32 | -26 | 13 | 0.57 | 0.51 |
|  | 2 | 65 | -33 | 20 | 0.45 | 0.38 |
|  | 3 | 58 | -16 | 7 | 0.37 | 0.32 |
|  | 4 | -38 | -33 | 17 | 0.49 | 0.43 |
|  | 5 | -60 | -25 | 14 | 0.46 | 0.39 |
|  | 6 | -49 | -26 | 5 | 0.42 | 0.36 |
|  | 7 | 43 | -23 | 20 | 0.57 | 0.51 |
|  | 8 | -50 | -34 | 26 | 0.50 | 0.44 |
|  | 9 | -53 | -22 | 23 | 0.56 | 0.50 |
|  | 10 | -55 | -9 | 12 | 0.47 | 0.43 |
|  | 11 | 56 | -5 | 13 | 0.37 | 0.33 |
|  | 12 | 59 | -17 | 29 | 0.52 | 0.46 |
|  | 13 | -30 | -27 | 12 | 0.44 | 0.38 |
| DMN |  |  |  |  |  |  |
|  | 1 | -41 | -75 | 26 | 0.59 | 0.53 |
|  | 2 | 6 | 67 | -4 | 0.48 | 0.41 |
|  | 3 | 8 | 48 | -15 | 0.60 | 0.54 |
|  | 4 | -13 | -40 | 1 | 0.52 | 0.47 |
|  | 5 | -18 | 63 | -9 | 0.57 | 0.52 |
|  | 6 | -46 | -61 | 21 | 0.47 | 0.41 |
|  | 7 | 43 | -72 | 28 | 0.48 | 0.41 |
|  | 8 | -44 | 12 | -34 | 0.47 | 0.41 |
|  | 9 | 46 | 16 | -30 | 0.57 | 0.52 |
|  | 10 | -68 | -23 | -16 | 0.60 | 0.55 |
|  | 11 | -44 | -65 | 35 | 0.36 | 0.33 |
|  | 12 | -39 | -75 | 44 | 0.40 | 0.36 |
|  | 13 | -7 | -55 | 27 | 0.36 | 0.33 |
|  | 14 | 6 | -59 | 35 | 0.38 | 0.35 |
|  | 15 | -11 | -56 | 16 | 0.52 | 0.48 |
|  | 16 | -3 | -49 | 13 | 0.41 | 0.36 |
|  | 17 | 8 | -48 | 31 | 0.43 | 0.41 |
|  | 18 | 15 | -63 | 26 | 0.38 | 0.33 |
|  | 19 | -2 | -37 | 44 | 0.55 | 0.49 |
|  | 20 | 11 | -54 | 17 | 0.48 | 0.44 |
|  | 21 | 52 | -59 | 36 | 0.38 | 0.34 |
|  | 22 | 23 | 33 | 48 | 0.55 | 0.51 |
|  | 23 | -10 | 39 | 52 | 0.41 | 0.36 |
|  | 24 | -16 | 29 | 53 | 0.41 | 0.36 |
|  | 25 | -35 | 20 | 51 | 0.57 | 0.52 |
|  | 26 | 22 | 39 | 39 | 0.45 | 0.41 |
|  | 27 | 13 | 55 | 38 | 0.56 | 0.51 |
|  | 28 | -10 | 55 | 39 | 0.40 | 0.36 |
|  | 29 | -20 | 45 | 39 | 0.55 | 0.51 |
|  | 30 | 6 | 54 | 16 | 0.41 | 0.36 |
|  | 31 | 6 | 64 | 22 | 0.42 | 0.36 |
|  | 32 | -7 | 51 | -1 | 0.38 | 0.34 |
|  | 33 | 9 | 54 | 3 | 0.37 | 0.34 |
|  | 34 | -3 | 44 | -9 | 0.38 | 0.34 |
|  | 35 | 8 | 42 | -5 | 0.59 | 0.55 |
|  | 36 | -11 | 45 | 8 | 0.46 | 0.41 |
|  | 37 | -2 | 38 | 36 | 0.44 | 0.39 |
|  | 38 | -3 | 42 | 16 | 0.50 | 0.46 |
|  | 39 | -20 | 64 | 19 | 0.53 | 0.48 |
|  | 40 | -8 | 48 | 23 | 0.42 | 0.37 |
|  | 41 | 65 | -12 | -19 | 0.47 | 0.41 |
|  | 42 | -56 | -13 | -10 | 0.51 | 0.46 |
|  | 43 | -58 | -30 | -4 | 0.48 | 0.42 |
|  | 44 | 65 | -31 | -9 | 0.50 | 0.44 |
|  | 45 | -68 | -41 | -5 | 0.52 | 0.46 |
|  | 46 | 13 | 30 | 59 | 0.50 | 0.43 |
|  | 47 | 12 | 36 | 20 | 0.48 | 0.44 |
|  | 48 | 52 | -2 | -16 | 0.69 | 0.65 |
|  | 49 | -26 | -40 | -8 | 0.51 | 0.47 |
|  | 50 | 27 | -37 | -13 | 0.56 | 0.49 |
|  | 51 | -34 | -38 | -16 | 0.49 | 0.44 |
|  | 52 | 28 | -77 | -32 | 0.45 | 0.41 |
|  | 53 | 52 | 7 | -30 | 0.54 | 0.48 |
|  | 54 | -53 | 3 | -27 | 0.40 | 0.35 |
|  | 55 | 47 | -50 | 29 | 0.47 | 0.43 |
|  | 56 | -49 | -42 | 1 | 0.48 | 0.42 |
|  | 57 | -46 | 31 | -13 | 0.43 | 0.36 |
|  | 58 | 49 | 35 | -12 | 0.52 | 0.44 |
| VN |  |  |  |  |  |  |
|  | 1 | 18 | -47 | -10 | 0.51 | 0.46 |
|  | 2 | 40 | -72 | 14 | 0.54 | 0.49 |
|  | 3 | 8 | -72 | 11 | 0.29 | 0.26 |
|  | 4 | -8 | -81 | 7 | 0.39 | 0.36 |
|  | 5 | -28 | -79 | 19 | 0.54 | 0.50 |
|  | 6 | 20 | -66 | 2 | 0.40 | 0.37 |
|  | 7 | -24 | -91 | 19 | 0.51 | 0.47 |
|  | 8 | 27 | -59 | -9 | 0.44 | 0.40 |
|  | 9 | -15 | -72 | -8 | 0.44 | 0.40 |
|  | 10 | -18 | -68 | 5 | 0.37 | 0.34 |
|  | 11 | 43 | -78 | -12 | 0.46 | 0.41 |
|  | 12 | -47 | -76 | -10 | 0.51 | 0.45 |
|  | 13 | -14 | -91 | 31 | 0.43 | 0.40 |
|  | 14 | 15 | -87 | 37 | 0.40 | 0.36 |
|  | 15 | 29 | -77 | 25 | 0.43 | 0.40 |
|  | 16 | 20 | -86 | -2 | 0.57 | 0.51 |
|  | 17 | 15 | -77 | 31 | 0.25 | 0.21 |
|  | 18 | -16 | -52 | -1 | 0.45 | 0.40 |
|  | 19 | 42 | -66 | -8 | 0.46 | 0.41 |
|  | 20 | 24 | -87 | 24 | 0.36 | 0.34 |
|  | 21 | 6 | -72 | 24 | 0.47 | 0.42 |
|  | 22 | -42 | -74 | 0 | 0.47 | 0.42 |
|  | 23 | 26 | -79 | -16 | 0.45 | 0.41 |
|  | 24 | -16 | -77 | 34 | 0.51 | 0.46 |
|  | 25 | -3 | -81 | 21 | 0.35 | 0.31 |
|  | 26 | -40 | -88 | -6 | 0.44 | 0.39 |
|  | 27 | 37 | -84 | 13 | 0.48 | 0.45 |
|  | 28 | 6 | -81 | 6 | 0.34 | 0.30 |
|  | 29 | -26 | -90 | 3 | 0.56 | 0.51 |
|  | 30 | -33 | -79 | -13 | 0.49 | 0.44 |
|  | 31 | 37 | -81 | 1 | 0.56 | 0.51 |
| FPN |  |  |  |  |  |  |
|  | 1 | -44 | 2 | 46 | 0.52 | 0.45 |
|  | 2 | 48 | 25 | 27 | 0.54 | 0.47 |
|  | 3 | -47 | 11 | 23 | 0.54 | 0.46 |
|  | 4 | -53 | -49 | 43 | 0.57 | 0.51 |
|  | 5 | -23 | 11 | 64 | 0.64 | 0.59 |
|  | 6 | 58 | -53 | -14 | 0.55 | 0.47 |
|  | 7 | 24 | 45 | -15 | 0.68 | 0.62 |
|  | 8 | 34 | 54 | -13 | 0.59 | 0.54 |
|  | 9 | 47 | 10 | 33 | 0.53 | 0.46 |
|  | 10 | -41 | 6 | 33 | 0.54 | 0.49 |
|  | 11 | -42 | 38 | 21 | 0.54 | 0.48 |
|  | 12 | 38 | 43 | 15 | 0.58 | 0.51 |
|  | 13 | 49 | -42 | 45 | 0.57 | 0.51 |
|  | 14 | -28 | -58 | 48 | 0.58 | 0.53 |
|  | 15 | 44 | -53 | 47 | 0.44 | 0.40 |
|  | 16 | 32 | 14 | 56 | 0.47 | 0.42 |
|  | 17 | 37 | -65 | 40 | 0.47 | 0.42 |
|  | 18 | -42 | -55 | 45 | 0.53 | 0.48 |
|  | 19 | 40 | 18 | 40 | 0.53 | 0.47 |
|  | 20 | -34 | 55 | 4 | 0.64 | 0.58 |
|  | 21 | -42 | 45 | -2 | 0.56 | 0.49 |
|  | 22 | 33 | -53 | 44 | 0.61 | 0.57 |
|  | 23 | 43 | 49 | -2 | 0.61 | 0.54 |
|  | 24 | -42 | 25 | 30 | 0.62 | 0.54 |
|  | 25 | -3 | 26 | 44 | 0.55 | 0.49 |
| SAN |  |  |  |  |  |  |
|  | 1 | 11 | -39 | 50 | 0.66 | 0.59 |
|  | 2 | 55 | -45 | 37 | 0.56 | 0.50 |
|  | 3 | 42 | 0 | 47 | 0.51 | 0.44 |
|  | 4 | 31 | 33 | 26 | 0.56 | 0.50 |
|  | 5 | 48 | 22 | 10 | 0.49 | 0.43 |
|  | 6 | -35 | 20 | 0 | 0.48 | 0.43 |
|  | 7 | 36 | 22 | 3 | 0.42 | 0.39 |
|  | 8 | 37 | 32 | -2 | 0.53 | 0.47 |
|  | 9 | 34 | 16 | -8 | 0.53 | 0.49 |
|  | 10 | -11 | 26 | 25 | 0.45 | 0.39 |
|  | 11 | -1 | 15 | 44 | 0.43 | 0.38 |
|  | 12 | -28 | 52 | 21 | 0.56 | 0.49 |
|  | 13 | 0 | 30 | 27 | 0.41 | 0.37 |
|  | 14 | 5 | 23 | 37 | 0.43 | 0.38 |
|  | 15 | 10 | 22 | 27 | 0.44 | 0.40 |
|  | 16 | 31 | 56 | 14 | 0.57 | 0.51 |
|  | 17 | 26 | 50 | 27 | 0.55 | 0.50 |
|  | 18 | -39 | 51 | 17 | 0.54 | 0.48 |
| SUB |  |  |  |  |  |  |
|  | 1 | 6 | -24 | 0 | 0.52 | 0.47 |
|  | 2 | -2 | -13 | 12 | 0.54 | 0.49 |
|  | 3 | -10 | -18 | 7 | 0.46 | 0.40 |
|  | 4 | 12 | -17 | 8 | 0.44 | 0.40 |
|  | 5 | -5 | -28 | -4 | 0.51 | 0.45 |
|  | 6 | -22 | 7 | -5 | 0.42 | 0.36 |
|  | 7 | -15 | 4 | 8 | 0.47 | 0.41 |
|  | 8 | 31 | -14 | 2 | 0.43 | 0.39 |
|  | 9 | 23 | 10 | 1 | 0.43 | 0.39 |
|  | 10 | 29 | 1 | 4 | 0.49 | 0.44 |
|  | 11 | -31 | -11 | 0 | 0.44 | 0.39 |
|  | 12 | 15 | 5 | 7 | 0.50 | 0.45 |
|  | 13 | 9 | -4 | 6 | 0.67 | 0.61 |
| VAN |  |  |  |  |  |  |
|  | 1 | -10 | 11 | 67 | 0.59 | 0.53 |
|  | 2 | 54 | -43 | 22 | 0.49 | 0.43 |
|  | 3 | -56 | -50 | 10 | 0.46 | 0.41 |
|  | 4 | -55 | -40 | 14 | 0.47 | 0.41 |
|  | 5 | 52 | -33 | 8 | 0.55 | 0.49 |
|  | 6 | 51 | -29 | -4 | 0.57 | 0.50 |
|  | 7 | 56 | -46 | 11 | 0.49 | 0.43 |
|  | 8 | 53 | 33 | 1 | 0.55 | 0.49 |
|  | 9 | -49 | 25 | -1 | 0.55 | 0.48 |
| DAN |  |  |  |  |  |  |
|  | 1 | 10 | -62 | 61 | 0.51 | 0.45 |
|  | 2 | -52 | -63 | 5 | 0.63 | 0.56 |
|  | 3 | 22 | -65 | 48 | 0.54 | 0.49 |
|  | 4 | 46 | -59 | 4 | 0.58 | 0.52 |
|  | 5 | 25 | -58 | 60 | 0.49 | 0.44 |
|  | 6 | -33 | -46 | 47 | 0.61 | 0.55 |
|  | 7 | -27 | -71 | 37 | 0.61 | 0.54 |
|  | 8 | -32 | -1 | 54 | 0.66 | 0.60 |
|  | 9 | -42 | -60 | -9 | 0.59 | 0.54 |
|  | 10 | -17 | -59 | 64 | 0.62 | 0.57 |
|  | 11 | 29 | -5 | 54 | 0.63 | 0.57 |
| UND |  |  |  |  |  |  |
|  | 1 | -25 | -98 | -12 | 0.65 | 0.58 |
|  | 2 | 27 | -97 | -13 | 0.64 | 0.56 |
|  | 3 | 24 | 32 | -18 | 0.61 | 0.54 |
|  | 4 | -56 | -45 | -24 | 0.63 | 0.57 |
|  | 5 | 8 | 41 | -24 | 0.70 | 0.63 |
|  | 6 | -21 | -22 | -20 | 0.67 | 0.62 |
|  | 7 | 17 | -28 | -17 | 0.70 | 0.69 |
|  | 8 | -37 | -29 | -26 | 0.62 | 0.53 |
|  | 9 | 65 | -24 | -19 | 0.38 | 0.31 |
|  | 10 | 52 | -34 | -27 | 0.53 | 0.46 |
|  | 11 | 55 | -31 | -17 | 0.54 | 0.46 |
|  | 12 | 34 | 38 | -12 | 0.70 | 0.63 |
|  | 13 | -58 | -26 | -15 | 0.55 | 0.49 |
|  | 14 | 27 | 16 | -17 | 0.62 | 0.55 |
|  | 15 | -31 | 19 | -19 | 0.61 | 0.55 |
|  | 16 | -2 | -35 | 31 | 0.47 | 0.44 |
|  | 17 | -7 | -71 | 42 | 0.60 | 0.54 |
|  | 18 | 11 | -66 | 42 | 0.51 | 0.46 |
|  | 19 | 4 | -48 | 51 | 0.50 | 0.43 |
|  | 20 | 8 | -91 | -7 | 0.48 | 0.43 |
|  | 21 | 17 | -91 | -14 | 0.53 | 0.47 |
|  | 22 | -12 | -95 | -13 | 0.62 | 0.57 |
|  | 23 | -21 | 41 | -20 | 0.74 | 0.68 |
|  | 24 | -18 | -76 | -24 | 0.52 | 0.45 |
|  | 25 | 17 | -80 | -34 | 0.47 | 0.42 |
|  | 26 | 35 | -67 | -34 | 0.55 | 0.47 |
|  | 27 | 2 | -24 | 30 | 0.59 | 0.55 |
|  | 28 | -16 | -65 | -20 | 0.58 | 0.52 |
|  | 29 | -32 | -55 | -25 | 0.54 | 0.47 |
|  | 30 | 22 | -58 | -23 | 0.66 | 0.60 |
|  | 31 | 1 | -62 | -18 | 0.59 | 0.52 |
|  | 32 | 33 | -12 | -34 | 0.66 | 0.59 |
|  | 33 | -31 | -10 | -36 | 0.62 | 0.55 |
|  | 34 | 49 | -3 | -38 | 0.64 | 0.58 |
|  | 35 | -50 | -7 | -39 | 0.55 | 0.48 |
|  | 36 | -47 | -51 | -21 | 0.63 | 0.56 |
|  | 37 | 46 | -47 | -17 | 0.58 | 0.51 |

SMN, somatosensory network; CO, cingulo-opercular network; AN, auditory network; DMN, default-mode network; VN, visual network; FPN, fronto-parietal network; SAN, salience network; SUB, subcortical network; VAN, ventral attention network; DAN, dorsal attention network; UND, uncertain network.

1. **Contribution of each functional network in predicting the severity of ASD symptoms**

The corresponding feature weights of the functional network in the brain-behavior prediction analysis are shown in Figure S7. The severity of social communication impairments in ASD can be predicted by IDFC of ASD subtype 1, with higher weights for VN, VAN and DAN and lower weights for DMN, FPN and SUB. The severity of restricted and repetitive behaviors in ASD can be predicted by IDFC of ASD subtype 2, with higher weights for SMN, VN and DAN and lower weights for CO and SUB.


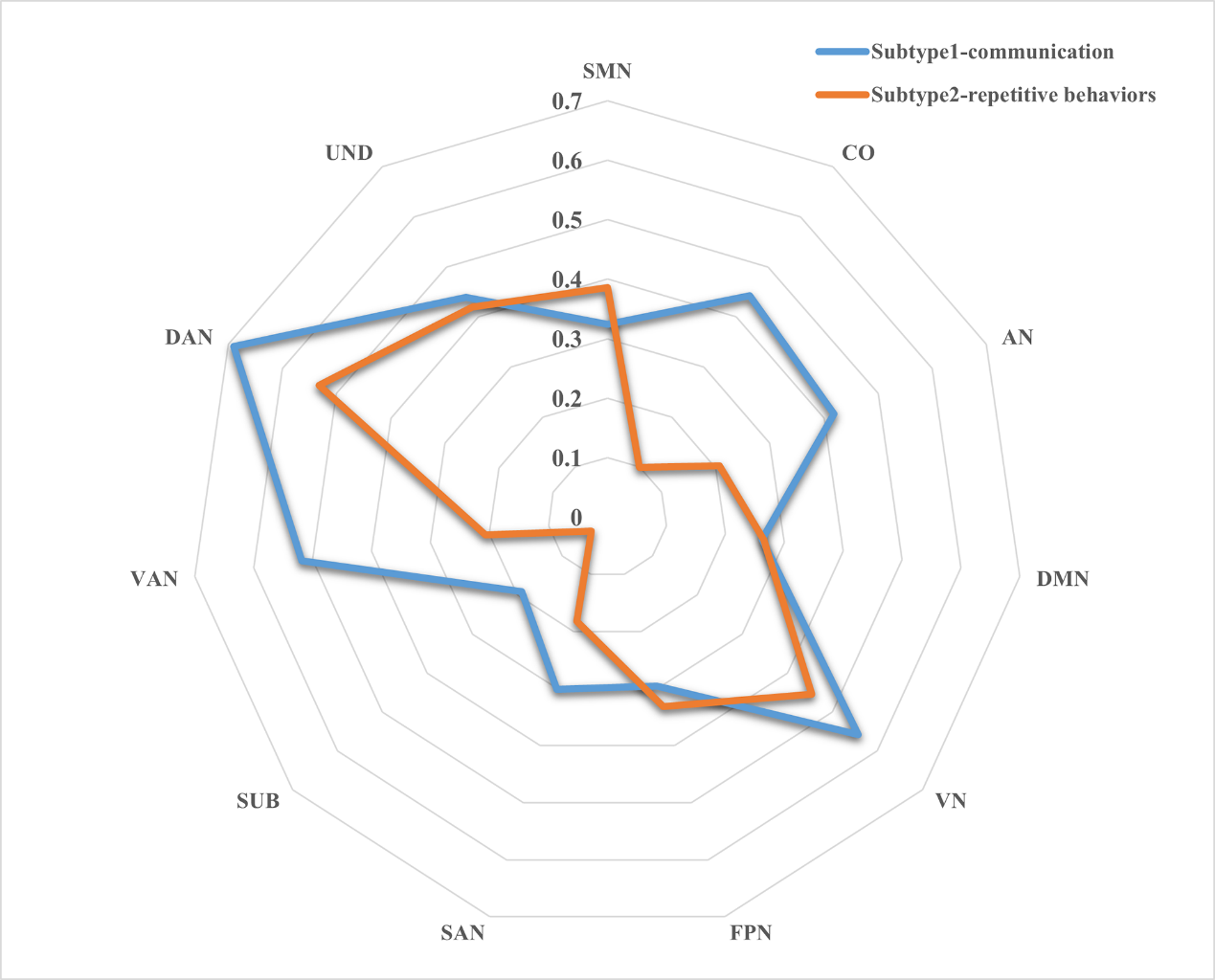


**Figure S7. Contribution of each functional network in predicting the severity of ASD symptoms.** SMN, somatosensory network; CO, cingulo-opercular network; AN, auditory network; DMN, default-mode network; VN, visual network; FPN, fronto-parietal network; SAN, salience network; SUB, subcortical network; VAN, ventral attention network; DAN, dorsal attention network; UND, uncertain network.

1. **Within-group inter-individual FC deviation differences between the ASD and TC groups**

**Table S3. Results of two-sample t-tests for within-group inter-individual FC deviation at the region level of ASD and TC groups.**

| Brain networks | Nodes | MNI coordinates | | | t |
| --- | --- | --- | --- | --- | --- |
|  |  | *x* | *y* | *z* |  |
| SMN |  |  |  |  |  |
|  | 1 | 13 | -33 | 75 | -4.12 |
|  | 2 | 10 | -17 | 74 | 3.65 |
| DMN |  |  |  |  |  |
|  | 1 | 6 | 67 | -4 | -2.88 |
|  | 2 | 46 | 16 | -30 | -3.62 |
|  | 3 | 52 | -59 | 36 | 3.92 |
|  | 4 | -2 | 38 | 36 | 2.98 |
|  | 5 | 12 | 36 | 20 | 3.04 |
|  | 6 | 28 | -77 | -32 | 3.32 |
|  | 7 | 47 | -50 | 29 | 3.87 |
| VN |  |  |  |  |  |
|  | 1 | 20 | -66 | 2 | 3.61 |
|  | 2 | 15 | -77 | 31 | 3.54 |
|  | 3 | -16 | -52 | -1 | 3.04 |
|  | 4 | 24 | -87 | 24 | 5.77 |
|  | 5 | -3 | -81 | 21 | 3.15 |
|  | 6 | 37 | -84 | 13 | 3.03 |
|  | 7 | 6 | -81 | 6 | 2.91 |
|  | 8 | 37 | -81 | 1 | 3.71 |
| FPN |  |  |  |  |  |
|  | 1 | 37 | -65 | 40 | 3.74 |
| SAN |  |  |  |  |  |
|  | 1 | 55 | -45 | 37 | 4.07 |
|  | 2 | 31 | 33 | 26 | 3.25 |
| SUB |  |  |  |  |  |
|  | 1 | -2 | -13 | 12 | 3.14 |
| VAN |  |  |  |  |  |
|  | 1 | 51 | -29 | -4 | -2.94 |
| UND |  |  |  |  |  |
|  | 1 | 17 | -28 | -17 | -2.95 |
|  | 2 | -7 | -71 | 42 | 3.03 |
|  | 3 | 17 | -80 | -34 | 4.14 |

SMN, somatosensory network; DMN, default-mode network; VN, visual network; FPN, fronto-parietal network; SAN, salience network; SUB, subcortical network; VAN, ventral attention network; UND, uncertain network.

1. **Within-group inter-individual FC deviation differences between the ASD group and TC subgroups**

We calculated the within-group inter-individual FC deviation at the region level for TC subgroup 1 and TC subgroup 2. The within-group inter-individual FC deviation differences at the region level between the two TC subgroups and the ASD group were analyzed separately using a two-sample t-test. The analysis process is the same as when using the whole TC group for analysis. The ASD group showed higher within-group inter-individual FC deviation at 20 ROIs and lower at 4 ROIs compared to the TC subgroup 1 (p < 0.05, FDR corrected), as shown in Figure S8A (see Table S4 for the standard table form of the results). Among them, the 20 ROIs with higher within-group inter-individual FC deviation include: one in DMN, fifteen in VN, one in FPN, one in VAN, one in DAN and one in UND. The 4 ROIs with lower within-group inter-individual FC deviation included one in SMN, one in VAN and two in UND. The ASD group showed higher within-group inter-individual FC deviation at 28 ROIs and lower at 2 ROIs compared to the TC subgroup 2 (p < 0.05, FDR corrected), as shown in Figure S8B (see Table S5 for the standard table form of the results). Among them, the 28 ROIs with higher within-group inter-individual FC deviation include: two in SMN, twelve in DMN, three in VN, three in FPN, one in SAN, one in SUB and six in UND. The 2 ROIs with lower within-group inter-individual FC deviation included one in DMN and one in UND.


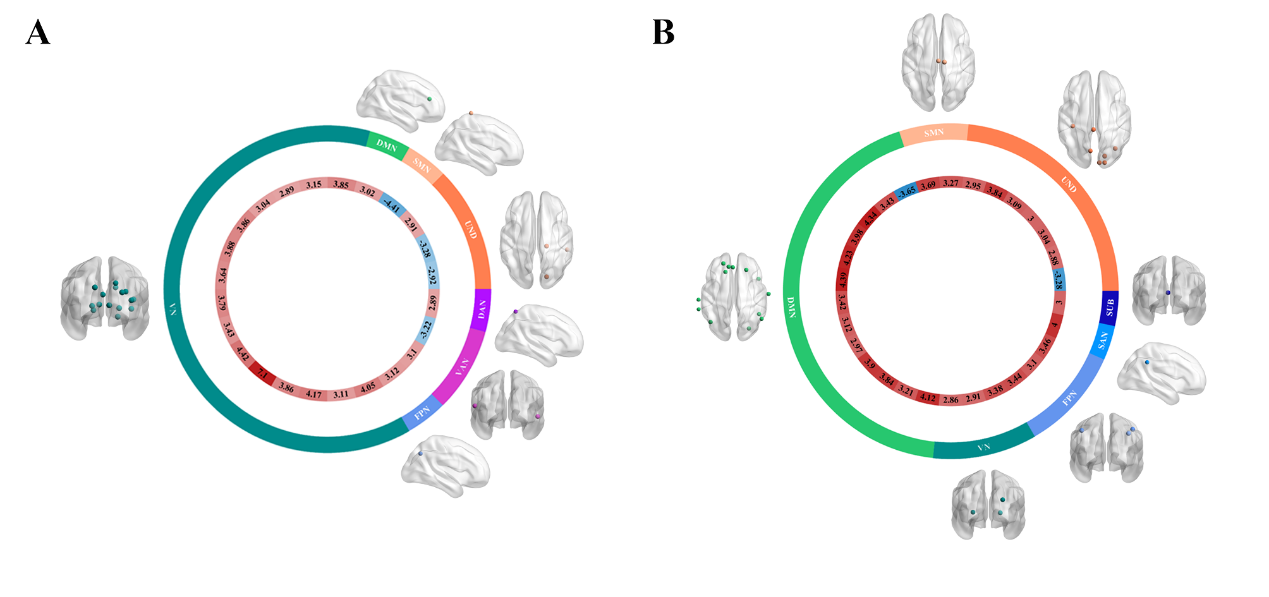


**Figure S8. Results of two-sample t-tests for within-group inter-individual FC deviation at the region level of the ASD group and TC subgroups.** The brain map shows the location of the ROIs. The outer circle indicates the network where the ROIs are located. The inner circle indicates the t-value of the two-sample t-tests (p < 0.05, FDR corrected). SMN, somatosensory network; DMN, default-mode network; VN, visual network; FPN, fronto-parietal network; SAN, salience network; SUB, subcortical network; VAN, ventral attention network; DAN, dorsal attention network; UND, uncertain network.

**Table S4. Results of two-sample t-tests for within-group inter-individual FC deviation at the region level of ASD and TC subgroup 1.**

| Brain networks | Nodes | MNI coordinates | | | t |
| --- | --- | --- | --- | --- | --- |
|  |  | *x* | *y* | *z* |  |
| SMN |  |  |  |  |  |
|  | 1 | 13 | -33 | 75 | -4.41 |
| DMN |  |  |  |  |  |
|  | 1 | 12 | 36 | 20 | 3.02 |
| VN |  |  |  |  |  |
|  | 1 | 18 | -47 | -10 | 3.85 |
|  | 2 | 40 | -72 | 14 | 3.15 |
|  | 3 | -8 | -81 | 7 | 2.89 |
|  | 4 | 20 | -66 | 2 | 3.04 |
|  | 5 | -18 | -68 | 5 | 3.86 |
|  | 6 | -14 | -91 | 31 | 3.88 |
|  | 7 | 15 | -87 | 37 | 3.64 |
|  | 8 | 29 | -77 | 25 | 3.79 |
|  | 9 | 15 | -77 | 31 | 3.43 |
|  | 10 | -16 | -52 | -1 | 4.42 |
|  | 11 | 24 | -87 | 24 | 7.10 |
|  | 12 | -3 | -81 | 21 | 3.86 |
|  | 13 | 37 | -84 | 13 | 4.17 |
|  | 14 | 6 | -81 | 6 | 3.11 |
|  | 15 | 37 | -81 | 1 | 4.05 |
| FPN |  |  |  |  |  |
|  | 1 | 37 | -65 | 40 | 3.12 |
| VAN |  |  |  |  |  |
|  | 1 | -55 | -40 | 14 | 3.10 |
|  | 2 | 51 | -29 | -4 | -3.22 |
| DAN |  |  |  |  |  |
|  | 1 | 10 | -62 | 61 | 2.89 |
| UND |  |  |  |  |  |
|  | 1 | 17 | -28 | -17 | -2.92 |
|  | 2 | 52 | -34 | -27 | -3.28 |
|  | 3 | 17 | -80 | -34 | 2.91 |

SMN, somatosensory network; DMN, default-mode network; VN, visual network; FPN, fronto-parietal network; VAN, ventral attention network; DAN, dorsal attention network; UND, uncertain network.

**Table S5. Results of two-sample t-tests for within-group inter-individual FC deviation at the region level of ASD and TC subgroup 2.**

| Brain networks | Nodes | MNI coordinates | | | t |
| --- | --- | --- | --- | --- | --- |
|  |  | *x* | *y* | *z* |  |
| SMN |  |  |  |  |  |
|  | 1 | 0 | -15 | 47 | 3.27 |
|  | 2 | 10 | -17 | 74 | 3.69 |
| DMN |  |  |  |  |  |
|  | 1 | 46 | 16 | -30 | -3.65 |
|  | 2 | -68 | -23 | -16 | 3.43 |
|  | 3 | -44 | -65 | 35 | 4.34 |
|  | 4 | 52 | -59 | 36 | 3.98 |
|  | 5 | 23 | 33 | 48 | 4.23 |
|  | 6 | -10 | 39 | 52 | 4.39 |
|  | 7 | -16 | 29 | 53 | 3.42 |
|  | 8 | -20 | 45 | 39 | 3.12 |
|  | 9 | -2 | 38 | 36 | 2.97 |
|  | 10 | 65 | -12 | -19 | 3.90 |
|  | 11 | -68 | -41 | -5 | 3.84 |
|  | 12 | 28 | -77 | -32 | 3.21 |
|  | 13 | 47 | -50 | 29 | 4.12 |
| VN |  |  |  |  |  |
|  | 1 | 20 | -66 | 2 | 2.86 |
|  | 2 | 24 | -87 | 24 | 2.91 |
|  | 3 | -26 | -90 | 3 | 3.38 |
| FPN |  |  |  |  |  |
|  | 1 | 44 | -53 | 47 | 3.44 |
|  | 2 | 37 | -65 | 40 | 3.10 |
|  | 3 | -42 | -55 | 45 | 3.46 |
| SAN |  |  |  |  |  |
|  | 1 | 55 | -45 | 37 | 4.00 |
| SUB |  |  |  |  |  |
|  | 1 | -2 | -13 | 12 | 3.00 |
| UND |  |  |  |  |  |
|  | 1 | -37 | -29 | -26 | -3.28 |
|  | 2 | -2 | -35 | 31 | 2.88 |
|  | 3 | -7 | -71 | 42 | 3.04 |
|  | 4 | 8 | -91 | -7 | 3.00 |
|  | 5 | 17 | -91 | -14 | 3.09 |
|  | 6 | 17 | -80 | -34 | 3.84 |
|  | 7 | 35 | -67 | -34 | 2.95 |

SMN, somatosensory network; DMN, default-mode network; VN, visual network; FPN, fronto-parietal network; SAN, salience network; SUB, subcortical network; UND, uncertain network.

1. **Retest results using correlation matrix without removing negative correlation**

We repeated the analysis process using both the positive and negative FC, and reconstructed the IDFC at the network level of the ASD group as clustering features. Two ASD subtypes (33 subjects in subtype 1 and 72 subjects in subtype 2) were obtained by k-means clustering. No significant differences were found between ASD subtype 1 and ASD subtype 2 in terms of demographics (i.e., age, FIQ, handedness, eye status and mean FD). And no significant differences were found between the two ASD subtype groups in terms of clinical symptom severity by comparing ADOS subscores either. The clustering results were not affected by the source of the subjects (i.e., sites).

The ASD subtype 1, ASD subtype 2 and TC group network-level FC (including intra-network connectivity and inter-network connectivity) were compared. The subtype 1 group showed a significant decrease in FC on all 66 connectivity edges compared to the TC group, the subtype 2 group showed a significant increase in FC on 51 connectivity edges compared to the TC group, and the subtype 2 group showed a significant increase in FC on all 66 connectivity edges compared to the subtype 1 group (p < 0.05, FDR corrected) (Figure S9).


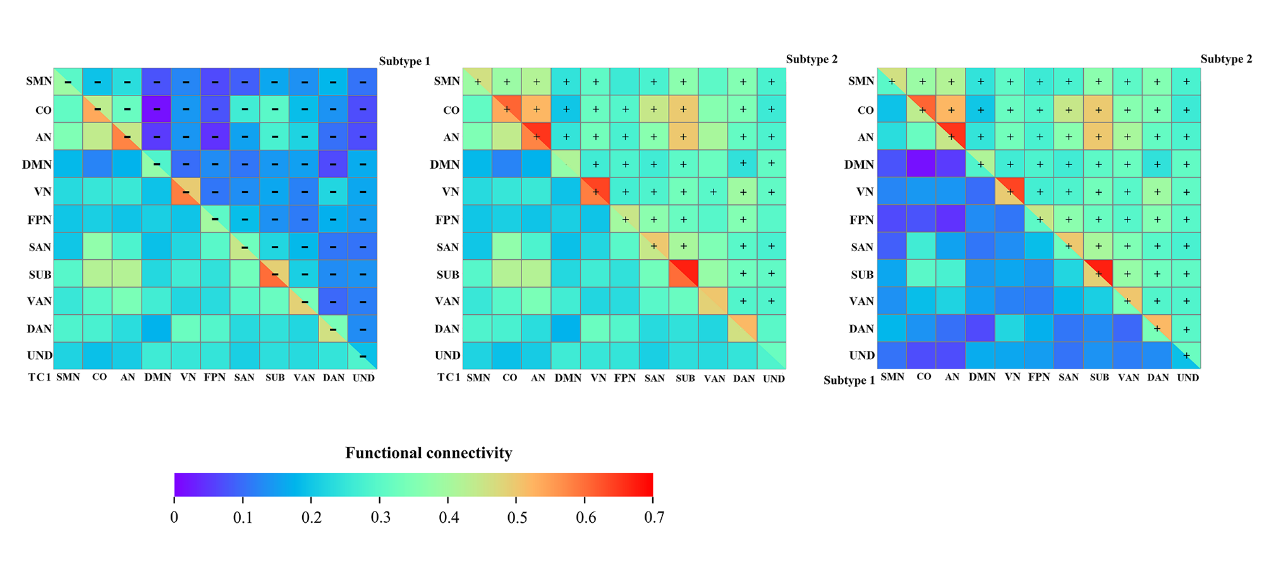


**Figure S9. Differences in FC of subtype 1, subtype 2 and TC at the network level in the analysis without removing negative FC.** The upper right and lower left triangles indicate the average network FC values for the different groups. +/- indicates a significant increase or decrease in FC for the group in the upper right compared to the group in the lower left (two-sample t-tests, p < 0.05, FDR corrected). SMN, somatosensory network; CO, cingulo-opercular network; AN, auditory network; DMN, default-mode network; VN, visual network; FPN, fronto-parietal network; SAN, salience network; SUB, subcortical network; VAN, ventral attention network; DAN, dorsal attention network; UND, uncertain network.

For each ASD subtype, the multivariate support vector regression model was used to investigate the relationship between IDFC and ASD symptom severity. Model performance was further assessed using LOOCV, and statistical significance was determined by a non-parametric permutation test. The network-level IDFC of ASD subtype 1 predicted the ADOS communication subscore (r = 0.33, p = 0.015; Figure S10A) and no significant relationship was observed with other subscores of ADOS. The network-level IDFC of ASD subtype 2 predicted the ADOS stereotypic behavior subscore (r = 0.32, p = 0.006; Figure S10B) and no significant relationship was observed with other subscores of ADOS.


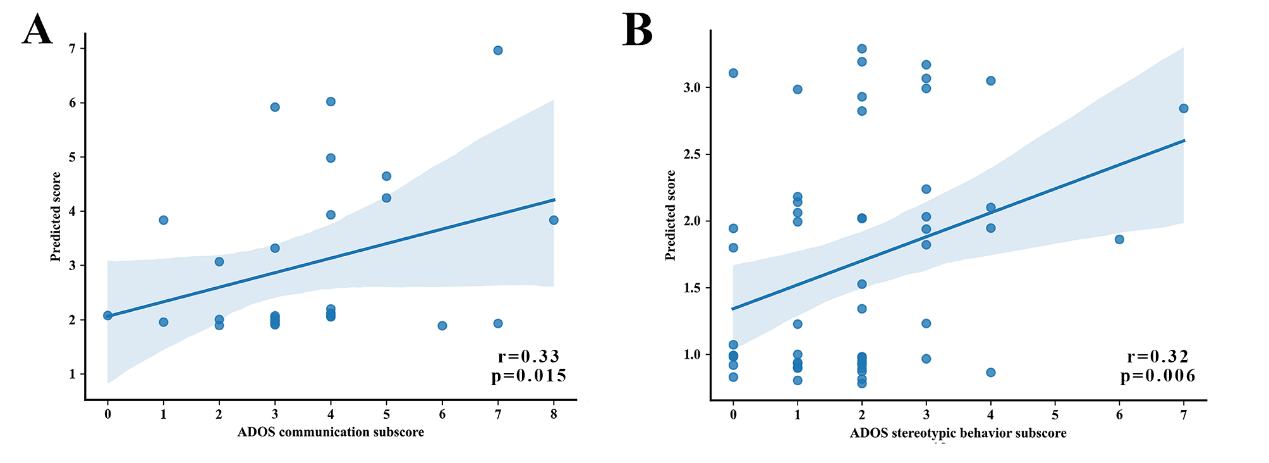


**Figure S10. Relationships between ADOS subscores and predicted scores using network-level IDFC in the analysis without removing negative FC.** (A) The relationship between ADOS communication subscore and predicted scores for ASD subtype 1. (B) The relationship between ADOS stereotypic behavior subscore and predicted scores for ASD subtype 2.

The inter-individual FC deviation matrix *V_aa__roi* for the ASD group and the inter-individual FC deviation matrix *V_tt__roi* for the TC group were reconstructed. The differences in within-group inter-individual FC deviation between the ASD and TC groups were analyzed from the ROI level using two-sample t-tests. Compared with the TC group, the ASD group showed higher within-group inter-individual FC deviation at 30 ROIs and lower at 5 ROIs (p < 0.05, FDR corrected), as shown in Figure S11. Among them, the 30 ROIs with higher within-group inter-individual FC deviation include: one in SMN, eleven in DMN, seven in VN, five in FPN, four in SAN, one in SUB and one in UND. The 5 ROIs with lower within-group inter-individual FC deviation included one in SMN, one in AN, one in DMN and two in UND. This result is also presented in table form as detailed in Table S6.


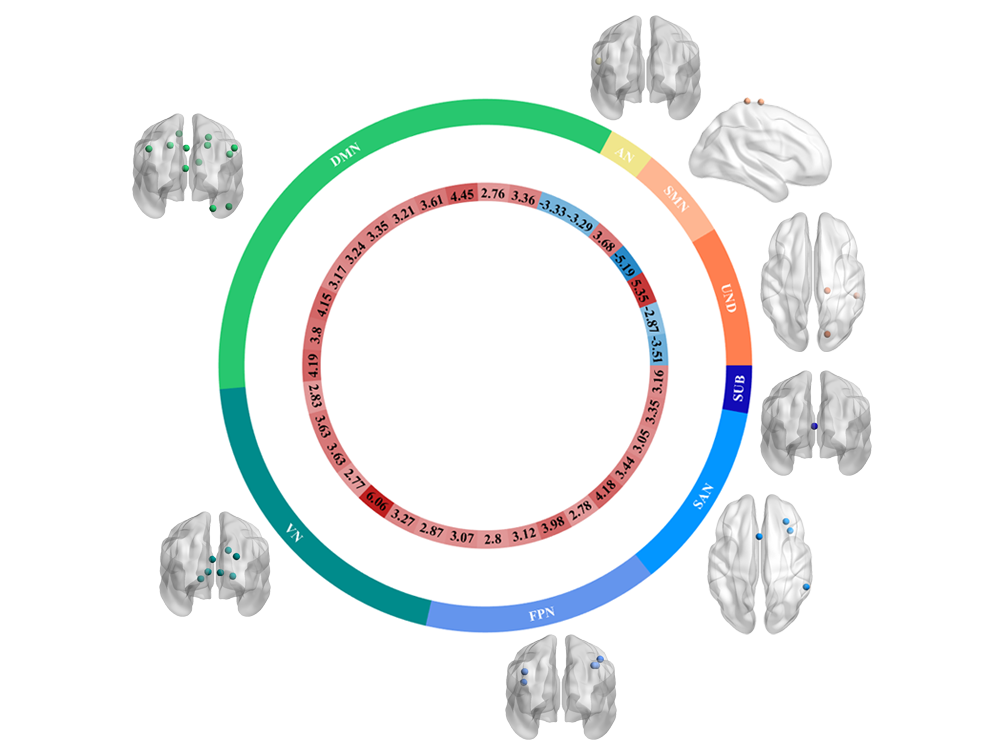


**Figure S11. Results of two-sample t-tests for within-group inter-individual FC deviation at the region level of ASD and TC groups in the analysis without removing negative FC.** The brain map shows the location of the ROIs (with significant differences between ASD and TC). The outer circle indicates the network where the ROIs are located. The inner circle indicates the t-value of the two-sample t-tests (p < 0.05, FDR corrected). SMN, somatosensory network; AN, auditory network; DMN, default-mode network; VN, visual network; FPN, fronto-parietal network; SAN, salience network; SUB, subcortical network; UND, uncertain network.

**Table S6. Results of two-sample t-tests for within-group inter-individual FC deviation at the region level of ASD and TC groups in the analysis without removing negative FC.**

| Brain networks | Nodes | MNI coordinates | | | t |
| --- | --- | --- | --- | --- | --- |
|  |  | *x* | *y* | *z* |  |
| SMN |  |  |  |  |  |
|  | 1 | 13 | -33 | 75 | -5.19 |
|  | 2 | 10 | -17 | 74 | 3.68 |
| AN |  |  |  |  |  |
|  | 1 | -53 | -22 | 23 | -3.29 |
| DMN |  |  |  |  |  |
|  | 1 | 46 | 16 | -30 | -3.33 |
|  | 2 | -44 | -65 | 35 | 3.36 |
|  | 3 | -3 | -49 | 13 | 2.76 |
|  | 4 | 52 | -59 | 36 | 4.45 |
|  | 5 | 23 | 33 | 48 | 3.61 |
|  | 6 | -10 | 39 | 52 | 3.21 |
|  | 7 | 22 | 39 | 39 | 3.35 |
|  | 8 | -20 | 45 | 39 | 3.24 |
|  | 9 | -2 | 38 | 36 | 3.17 |
|  | 10 | 12 | 36 | 20 | 4.15 |
|  | 11 | 28 | -77 | -32 | 3.80 |
|  | 12 | 47 | -50 | 29 | 4.19 |
| VN |  |  |  |  |  |
|  | 1 | -8 | -81 | 7 | 2.83 |
|  | 2 | 20 | -66 | 2 | 3.63 |
|  | 3 | 15 | -77 | 31 | 3.63 |
|  | 4 | -16 | -52 | -1 | 2.77 |
|  | 5 | 24 | -87 | 24 | 6.06 |
|  | 6 | -3 | -81 | 21 | 3.27 |
|  | 7 | 6 | -81 | 6 | 2.87 |
| FPN |  |  |  |  |  |
|  | 1 | -41 | 6 | 33 | 3.07 |
|  | 2 | -42 | 38 | 21 | 2.80 |
|  | 3 | 44 | -53 | 47 | 3.12 |
|  | 4 | 37 | -65 | 40 | 3.98 |
|  | 5 | 40 | 18 | 40 | 2.78 |
| SAN |  |  |  |  |  |
|  | 1 | 55 | -45 | 37 | 4.18 |
|  | 2 | 31 | 33 | 26 | 3.44 |
|  | 3 | 36 | 22 | 3 | 3.05 |
|  | 4 | -1 | 15 | 44 | 3.35 |
| SUB |  |  |  |  |  |
|  | 1 | -2 | -13 | 12 | 3.16 |
| UND |  |  |  |  |  |
|  | 1 | 17 | -28 | -17 | -3.51 |
|  | 2 | 52 | -34 | -27 | -2.87 |
|  | 3 | 17 | -80 | -34 | 5.35 |

SMN, somatosensory network; AN, auditory network; DMN, default-mode network; VN, visual network; FPN, fronto-parietal network; SAN, salience network; SUB, subcortical network; UND, uncertain network.
